# Nerves, nodes, and neoplasia

**DOI:** 10.3389/fimmu.2025.1674174

**Published:** 2025-09-15

**Authors:** Minjun Wang, Censhan Ran, Quan Liu

**Affiliations:** ^1^ Department of Biochemistry, Southern University of Science and Technology (SUSTech) Homeostatic Medicine Institute, University Laboratory of Metabolism and Health of Guangdong, Shenzhen, Guangdong, China; ^2^ Joint Laboratory of Guangdong-Hong Kong Universities for Vascular Homeostasis and Diseases, School of Medicine, Southern University of Science and Technology, Shenzhen, Guangdong, China; ^3^ School of Medicine, Shanghai Jiao Tong University, Shanghai, China

**Keywords:** lymph node, sympathetic nerve, nociceptor, tumor immunity, immunotherapy

## Abstract

Tumor immune evasion and incomplete responses to immunotherapy are some of the most significant obstacles in current cancer treatment. Since tumor-draining LNs (tdLNs) are cradles for anti-tumor immunity, and tumor-specific memory cells in tdLNs are the bona fide responders to immune-checkpoint blockade, tdLNs are increasingly valued in oncoimmunology research and cancer treatments. Recent progress has revealed that lymph nodes (LNs) are innervated and regulated by sensory and sympathetic nerve fibers. Because tumor cells, nerves, and immune cells coexist inside tdLNs—sites where anti-tumor immunity is initiated and compromised—it is critical to investigate whether tumor-neuro-immune crosstalk also occurs in these nodes. Although direct evidence in tdLNs is lacking, we synthesize emerging evidence supporting this possibility. We argue that validating this hypothesis will be essential for elucidating immune evasion mechanisms and advancing surgical and immunological strategies against tumors. In this review, we first introduce LN anatomy, highlighting its innervation by sensory and sympathetic fibers. We then examine the neural regulation of immune activities, especially those within LNs and those associated with a tumor context. We further discuss the multifaceted roles of tdLNs in tumor immunology, including orchestration of anti-tumor immunity and local immunosuppression, pre-metastatic LN remodeling, and induction of systemic tumor-specific immune tolerance. Furthermore, we look into tumor-neural interactions from two angles: tumor-induced nerve growth and activation, and neural regulation of tumor progression. Finally, we propose potential tumor-neuro-immune interactions in tdLNs, discuss current perspectives on LN handling in cancer therapy, and discuss clinical implications of the progress summarized in this review.

## 1 Introduction

Lymph nodes (LNs) are kidney-shaped secondary immune organs where adaptive immunity is initiated. By constant filtration of lymph drained from local tissue sites, LNs detect exogenous and autologous immune stimuli and quickly generate antigen-specific immune responses (1). During such responses, naïve T and B lymphocytes circulating through LNs become activated, differentiate into effector or memory subtypes, and re-enter the circulation to exert various immune functions. Effector B cells, also named plasma cells, migrate to the bone marrow and secrete antigen-binding antibodies. Effector T cells, on the other hand, travel to peripheral sites of infection and function by direct cytotoxicity. Memory B and T cells circulate in the bloodstream, ready to respond to future immune challenges (2).

Impaired anti-tumor immunosurveillance is now widely recognized as a key factor in tumorigenesis, and tumor immune evasion is one of the biggest obstacles in cancer treatment (3). Against this background, tumor-draining lymph nodes (tdLNs), the initiation sites of anti-tumor adaptive immunity, are increasingly valued in oncoimmunology research. Notably, tdLNs are at the crossroads of anti-cancer immunity, cancer immune evasion, and cancer treatment (4). In the presence of tumors, tdLNs are cradles of both B- and T-cell-mediated anti-tumor immunity (5, 6). Conversely, tumors can alter the structure of tdLNs, influence immune and parenchymal cells within, and induce tumor-specific immune tolerance (7, 8). Emerging evidence also suggests that tumors can co-opt tdLNs to facilitate systemic immune suppression, potentially driving the development of distant metastasis (9). Furthermore, tdLNs hold significant clinical relevance. While lymphadenectomy was once a standard therapeutic approach, its benefits have become controversial (10, 11). In the era of immunotherapy, tdLNs are recognized as initiation sites of therapy-induced responses, and neoadjuvant therapies have shown clear advantages (12, 13). Novel strategies, such as tdLN-targeted immunotherapy, have also shown promising results (14, 15). Although tumor-specific immunosuppression in tdLNs has been well-characterized, the underlying mechanisms remain incompletely understood.

The peripheral neural system innervates a wide range of non-lymphoid tissues, such as the skin (16), airway, and gut (17); and lymphoid tissues including the bone marrow (18–20), thymus (21), and spleen (22), where immune responses are regulated by neuropeptides and neurotransmitters, including neuromedin U (NMU), calcitonin gene-related peptide (CGRP), vasoactive intestinal peptide (VIP), substance P (SP), and norepinephrine (NE) (23–25). Neuroscience and immunological studies have highlighted complicated tumor-neuro-immune interplays within cancers (26–28).

Recent progress has revealed innervation of LNs by sensory and sympathetic nerve fibers (29–31), which suggests the coexistence of tumor cells, nerves, and immune cells with the tdLNs, where anti-tumor immunity is initiated and compromised. Thus, a comprehensive understanding of tumor-neuro-immune crosstalk within tdLNs is critical for unravelling the mechanisms behind tumor immune evasion. This review focuses on the latest findings regarding LN innervation, neural regulation of immune activity, tumor-nerve interplay, and the potential tripartite tumor-neuro-LN interactions. We also discuss the potential clinical relevance of tdLNs in tumor surgical and immunological therapies.

## 2 An overview of lymph node innervation

**Figure 1:**
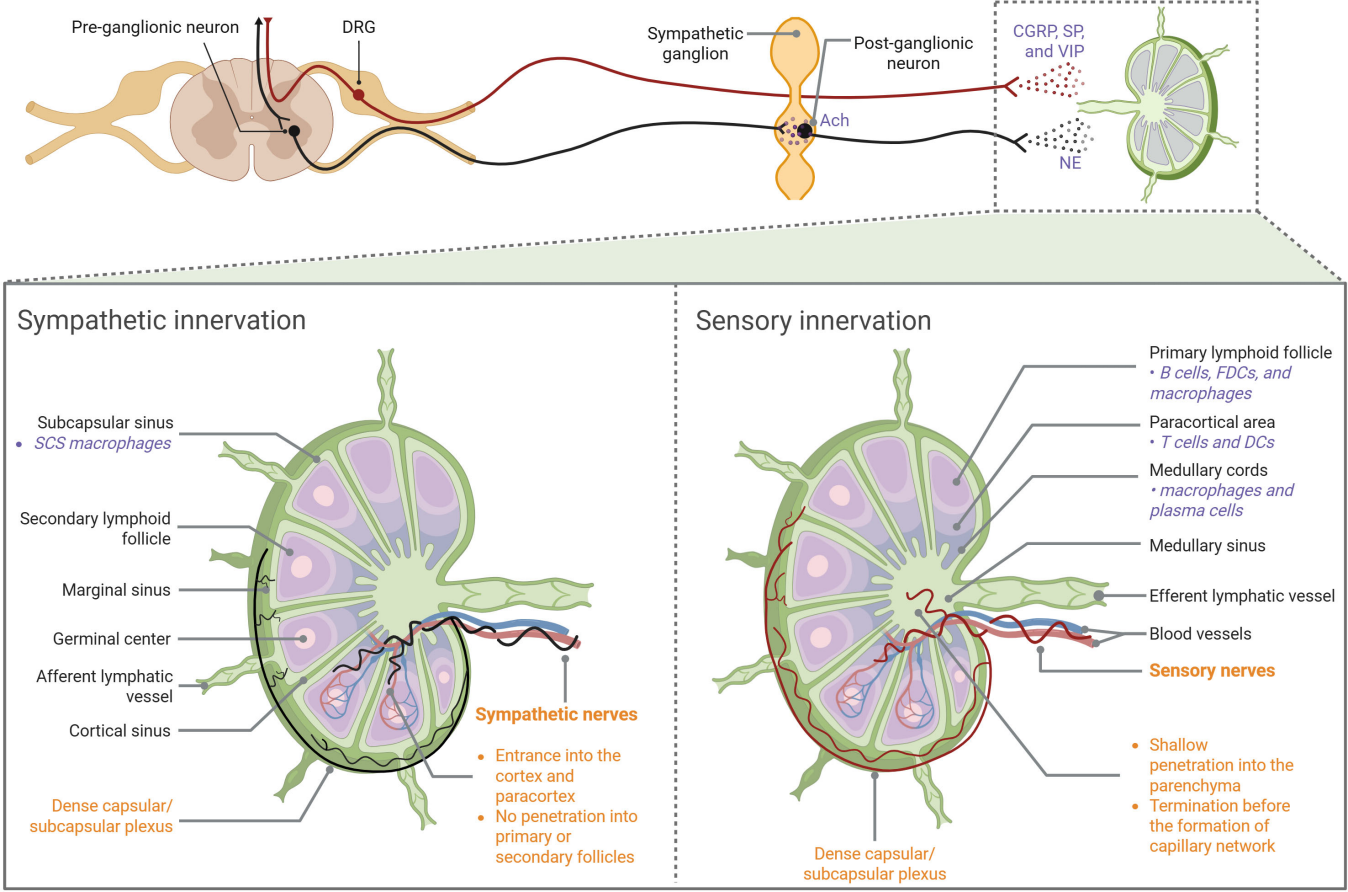
Anatomic overview of LN innervation Both sympathetic and sensory fibers enter LNs along blood vessels at the hilus region. Sympathetic nerves primarily innervate the vasculature, extending into the cortical and paracortical regions without penetrating the follicles or germinal centers. Sensory nerves not only aligned with blood vessels, but also branched extensively into the LN interstitial spaces. They penetrate shallow into the medulla but densely innervate the outer cortical region, forming a capsular/subcapsular network. CGRP, calcitonin gene-related peptide; DC, dendritic cell; DRG, dorsal root ganglia; FDC, follicular dendritic cell; NE, norepinephrine; SCS, subcapsular sinus; SP, substance P; VIP, vasoactive intestinal peptide.

LNs are anatomically composed of three regions: cortex, paracortex, and medulla. The parenchyma of an LN is composed of innate and adaptive immune cells, while the stroma provides structural support and houses blood vessels, maintaining the integrity of the node (1). LN innervation, a special stromal component that facilitates bidirectional neuro-immune crosstalk, was first characterized by Felten et al. in the 1980s (31). The field remained relatively quiet until advanced tracing and imaging techniques allowed for a more detailed characterization of LN anatomy and its dense innervation by both sympathetic and sensory fibers (**Figure 1**). The facts that 6-hydroxydopamine (6-OHDA)-mediated chemical sympathectomy does not alter sensory innervation and diphtheria toxin A (DTA)-mediated genetic ablation of sensory neurons does not affect TH^+^ fibers indicate that sympathetic and sensory innervation in LNs were mutually independent (30). There is a lack of definitive evidence of parasympathetic innervation inside LNs (30), although acetylcholine (Ach)-producing cells were identified (32).

### 2.1 Sympathetic innervation

Adrenergic neurons signal by releasing NE, which binds to α- or β-adrenergic receptors. As shown by catecholamine or tyrosine hydroxylase (TH) stain, sympathetic plexuses enter LNs in the hilar region and the fibers further extend into the parenchyma, wrapping around blood vessels (30, 33). TH^+^ fibers innervate the cortical and paracortical regions without reaching the follicles or germinal centers (GCs), while the subcapsular distribution of these fibers is controversial (30, 33).

### 2.2 Sensory innervation

The cell bodies of somatic sensory neurons are located in either the dorsal root ganglia (DRG) or the trigeminal ganglia (TG). When activated, these sensory neurons propagate action potentials to the central nervous system (CNS). However, upon reaching axonal branch points, these action potentials can also travel back to the peripheral nerve terminals, resulting in calcium influx and the rapid release of neuropeptides (30). Recent work used single-cell RNA-sequencing (RNA-seq) to reveal that LN-innervating sensory neurons mainly comprise four types of peptidergic nociceptors. Sensory fibers enter popliteal LNs at the hilus alongside arteries. However, unlike TH^+^ fibers, they not only aligned with blood vessels, but also branched extensively into the LN interstitial spaces. Some fibers penetrate the parenchyma and are located in the medulla, mostly terminating before arteries and arterioles branch into the capillary network. Other plexuses densely innervate the outer cortical region of the LNs, forming a capsular/subcapsular network. Fibers forming the network branch extensively in the LN capsule and extend into and below the subcapsular sinuses (SCSs), making contact with CD169^+^ SCS macrophages. Sensory fibers were rarely seen in the deep LN cortex and were absent from high endothelial venules (HEVs) (30).

## 3 Neural regulation of lymph node functions

**Figure 2:**
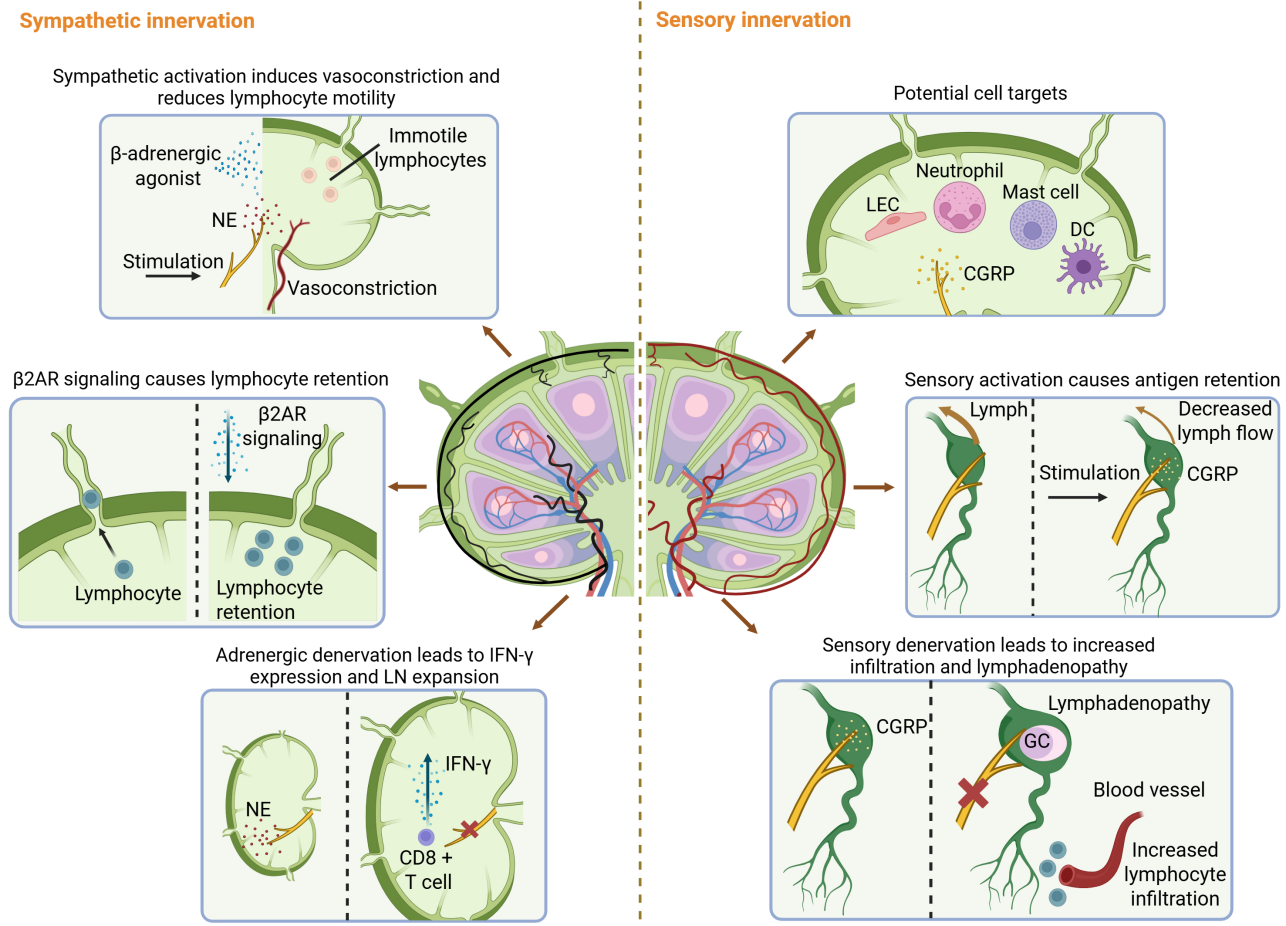
Neural regulation of LN functions. RNA-seq identified LECs, neutrophils, mast cells, and DCs as potential targets of sensory fibers in LNs. Sensory nerves modulate antigen flow and retention. Nociceptor activation results in restriction of antigen transit between LNs. Moreover, ablation of DRG neurons increased local immune infiltration and lymphadenopathy of the draining LN. Sympathetic innervation regulates LN immune responses. Sympathetic activation impairs anti-tumor immune responses within LNs and induces lymphocyte retention in LNs. Loss of direct adrenergic innervation induced the expression of IFN-γ in LN CD8^+^ T cells, which is responsible for LN expansion. β2AR, β2-adrenergic receptor; CGRP, calcitonin gene-related peptide; DC, dendritic cell; GC, germinal center; IFN-γ, interferon-gamma; LEC, lymphatic endothelial cell; NE, norepinephrine.

Nerve fibers that densely innervate LNs have been shown to play crucial roles in modulating LN functions (**Figure 2**). Huang et al. performed single-cell RNA-seq of LNs and DRG, and made ligand-receptor predictions based on cell-specific gene expression. Their analysis has identified lymphatic endothelial cells (LECs), neutrophils, mast cells, and dendritic cells (DCs) as potential targets of sensory fibers in LNs (30). Moreover, sensory nerves modulate antigen flow and retention (30, 34). Fragment crystallizable region receptor (FcR)-mediated antigen-specific activation of a subset of Nav1.8^+^TRPV1^−^ nociceptors resulted in restriction of antigen transit between LNs (34). Ablation of Nav1.8^+^ DRG neurons during bacterial infection increased the draining LN’s local immune infiltration and lymphadenopathy (16). Furthermore, sympathetic innervation regulates LN immune responses. Activation of sympathetic nerves, NE administration, and β-adrenergic receptor (β-AR) excitation have been shown to impair anti-tumor immune responses within LNs by reducing the motility of T cells, B cells, and antigen-presenting cells (APCs), as well as inducing vasoconstriction (35). Additionally,β2-AR signaling in lymphocytes can induce their retention in LNs via CXCR4/CCR7 while suppressing inflammation (36, 37). Loss of direct adrenergic innervation induced the expression of interferon-gamma (IFN-γ) in LN CD8^+^ T cells, which is responsible for LN expansion (38).

Accumulating evidence suggests that neural signals may also contribute to the development of LNs (detailed in (39)). Although validation is needed, studies on tertiary lymphoid structures (TLSs) may provide inspiration. LNs are closely resembled in formation, structure, and function by the transient lymphoid structures called TLSs (40). TLSs appear in sites with chronic inflammation, and GC-positive TLSs correlate with a favorable prognosis in most solid cancer types (40). It is now known that the formation of TLSs is under neural regulation. Sympathetic innervation facilitates TLS formation during acute lung inflammation (41), while sensory denervation in the skin enhances TLS formation in the melanoma tumor microenvironment (TME) and reduces tumor growth (42). Notably, a recent study shows that TLSs are induced by activated group 2 innate lymphoid cells (ILC2s) (43). Since CGRP can suppress ILC2 function (24), this study may explain the suppression of TLS formation by sensory nerves.

## 4 Tumor-draining lymph nodes in anti-cancer immunity and cancer immune evasion

Even before the formal concept of anti-tumor immunity emerged, physicians were already leveraging the immune system to fight malignancies (44). While the “cancer immunosurveillance” hypothesis proposed by Thomas and Burnet spurred extensive research into anti-cancer immunity (45), most of this research has focused on the TME. Instead, LNs had been viewed as passive organs susceptible to colonization by tumor cells (46). Clinical observations that LN involvement often preceded the occurrence of distant metastases in most solid organ malignancies led the 20th-century surgeon William Halsted to consider LN metastasis as a prerequisite for distant spread (47). However, molecular reconstruction of clonal phylogenies demonstrated that LN and distant metastases often arose from independent subclones within the primary tumor (48, 49), and tumor cells colonizing LNs were found to disseminate through LN blood vessels instead of efferent lymphatic vessels (50, 51). Rather than simply serving as passive staging posts during metastasis, accumulating evidence suggests that LNs have pivotal roles in anti-cancer immunity, cancer immune evasion, and cancer immunotherapy.

### 4.1 Tumor-draining lymph nodes are cradles for anti-tumor immunity and immunotherapy-induced responses

A widely accepted framework for anti-tumor immunity is the cancer-immunity cycle (CIC). In short, it holds that dying tumor cells release antigens, which are then captured by DCs and presented to T cells in the LNs. Activated T cells subsequently migrate to the tumor site, attack tumor cells, and release additional antigens, further driving this cycle (52). Recent advances suggest key revisions to the CIC model. First, while the conventional CIC model emphasizes CD8^+^ T cell-centered immunity, the roles of other immune cells in anti-tumor immunity are increasingly appreciated. CD4^+^ T cells and DCs are crucial maintainers of the cycle, while B cells may combat tumor cells via antibody production (53, 54). Second, T-cell mediated anti-tumor immunity comprises two phases, and the first phase occurs in tdLNs (5). Current opinions regarding tdLN-mediated anti-tumor immunity are reviewed below.

#### 4.1.1 CD8^+^ T cell responses

In acute infections, CD8^+^ T cells are primed in LNs to become fully-activated effector cells with antigen-specific cytotoxic functions (55). However, CD8^+^ T cell activation in cancer comprises an initial phase in tdLNs, followed by final differentiation within the tumor (5). One of the characteristics of chronic infections, including cancer, is that CD8^+^ T cells responding to antigenic stimulation would gradually lose their efficacy and become terminally exhausted T cells (Tex^term^ cells). They are characterized by persistent upregulation of inhibitory receptors and decreased secretion of effector molecules (56). Recent research indicated that Tex^term^ cells arose from a subset of T cell factor-1 (TCF-1) expressing CD8^+^ T cells called “progenitor exhausted” (Tpex) cells inside tdLNs. They robustly proliferate but have diminished cytotoxicity. Tpex cells from tdLNs constantly migrate to the tumor site (56). In the TME, when sufficient CD4^+^ T cells and APCs are present, Tpex cells initially differentiate into effector-like intermediate Tex cells (Tex^int^) with augmented effector cytotoxic functions, which then differentiate into Tex^term^ cells (57). The mechanisms behind the two-phase activation of tumor-specific CD8^+^ T cells are under investigation. One study linked T cell differentiation trajectory to tumor antigen dominance, revealing that T cells specific for “dominant” antigens that stably bind major histocompatibility complex (MHC) molecules were enriched for “effector” and “exhausted” T cell signatures. In contrast, recognizing subdominant antigens kept T cells in a TCF-1^+^ stem-like state (58). Another study demonstrated that the differentiation of naïve T cells into effector cells depends on their positioning at the periphery of the LN. In the tumor context, the lack of CXCR3 signaling restricts T cells to the center of the LN, inducing their differentiation into stem-like memory cell precursors (59).

Notably, TCF-1^+^ CD8^+^ T cells inside tdLNs establish memory-associated epigenetic program early in tumorigenesis, and are bona fide responders to programmed death 1 (PD-1)/programmed death ligand 1 (PD-L1) blockade (60, 61). Lineage tracing further revealed them as progenitors of tumor-specific exhausted CD8^+^ T cells in the TME after immune checkpoint blockade (ICB) (62, 63). A recent study demonstrated that LN-targeted chemoimmunotherapy can elicit stem-like CD8^+^ T cell responses, which evokes systemic tumor control (64). Interestingly, while TCF1 maintains CD8^+^ T cell stemness, a recent finding revealed that TCF1 is essential for CD8^+^ T cell priming and ICB response in poorly immunogenic tumors, but is dispensable in highly immunogenic tumors that efficiently expand transitory effectors (65).

#### 4.1.2 CD4^+^ T cell responses

Unlike CD8^+^ T cells, the roles of CD4^+^ T cells during carcinogenesis are still relatively unclear. However, studies have revealed that they act as crucial helpers of CD8^+^ T cells. In humans and mice, priming of tumor-specific CD4^+^ T cells primarily occurs in tdLNs (53). During the first anti-tumor CD8+ T cell activation phase, CD4^+^ T cells inside tdLNs “license” APCs for efficient cytotoxic lymphocyte priming and maintenance as memory cells (66). Meanwhile, CD4^+^ follicular helper T cells (Tfhs) provide help for tumor-specific B cell activation (67). During the second phase of CD8^+^ T cell activation, CD4^+^ T cells promote the accumulation, expansion, trafficking, and differentiation of tumor-specific CD8^+^ T cells within tumors (68). A recent study of triple-negative breast cancer found preferred T helper type 2 (Th2) cell polarization within tdLNs of “cold” (with <10% tumor infiltrating lymphocytes) tumors compared with “hot” (with >60% tumor infiltrating lymphocytes) tumors. Intriguingly, Th2/Th1 ratios in cold tumors were also significantly higher than in hot tumors, indicating coordination in Th subset differentiation between tdLNs and the TME (69).

CD4^+^ T cells in tdLNs also respond to immunotherapies. CD4^+^ naive/central-memory T cells from tdLN directly respond to anti-cytotoxic T lymphocyte antigen-4 (anti-CTLA4) therapy, trafficking via blood to the tumor, where they acquire a Th1 phenotype (70). In addition, anti-PD-1 therapy triggers Tfh cell-dependent interleukin (IL)-4 release that boosts CD8^+^ T cell responses in tdLNs (71). Interestingly, a recent study showed that the persistence of anti-tumor CD4^+^ T cells is critical for effective PD-1 and CTLA4-based cancer immunotherapies in elderly mice, as correcting DCs’ migratory defects in the elderly induced Th1 CD4^+^ T cells with cytolytic activity that drive anti-tumor immunity (72).

#### 4.1.3 Dendritic cell responses

Recent studies are increasingly focused on the roles of DCs in anti-cancer immunity and immunotherapy. Like CD4^+^ T cells, DCs also play crucial roles in both tumor-specific CD8^+^ T cell activation phases. DCs transport antigens from tumors to LNs and present them to T cells, facilitating their initial activation inside tdLNs (73). A recent study in melanoma showed that tumor antigens were primarily transferred to tdLNs by DCs instead of arriving as microparticles or exosomes. On arrival, tumor antigens are further transferred from migratory conventional type 1 DCs (cDC1s) and conventional type 2 DCs (cDC2s) to resident DCs (rDCs) via direct synaptic transfer (74). Similarly, cDCs activate T cells in the TME, which is positively associated with patient survival. Notably, although cDC2s and monocyte-derived DCs comprise most of the myeloid APCs in the TME (5), a recent study showed that tumor-specific T cells were primarily activated in the TME by cDC1s (75), which functioned simultaneously to support both CD8^+^ and CD4^+^ T cell priming (66).

DCs have recently been identified as essential orchestrators of anticancer immunotherapy (76). A high density of PD-1/PD-L1 interactions in tdLNs is associated with early disease relapse after surgery in patients with stage II melanoma, and these interactions occur mainly between T cells and DCs (77). In preclinical models of melanoma, anti-PD-1 antibodies increased CD5^+^ cDCs in both the TME and tdLNs via reduced production of IL-6, driving the activation of CD5^high^ CD4^+^ and CD8^+^ T cells (78). In addition, a recent study developed a bispecific DC-T cell engager that promoted the formation of DC-T cell crosstalk in the tumor and tdLNs, which enhanced the efficacy of anti-PD-1 immunotherapy (79). In mouse models, T cell immunoglobulin and mucin domain-containing-3 (TIM3) blockade reduced the number of tumor antigen-loaded cDCs in tdLNs, which impaired therapeutic effects (80).

#### 4.1.4 B cell responses

B cells and plasma cells inside tdLNs also have essential roles in shaping anti-tumor immune responses. GCs can be induced in tdLNs, with B cell expansion and increased antibody production (81). Although the anti-tumoral effects of antibodies produced by plasma cells primed in tdLNs are yet to be proven, antibodies derived from tumor-infiltrating B cells can directly fight against tumor cells. Tumor B-cell-derived IgA antagonizes the growth of ovarian cancer via tumor-antigen-specific and antigen-independent mechanisms (82). IgG produced by intratumoral plasma-like B cells can cause the degradation of target proteins inside tumor cells (83). Since B cells are rarely found on their own in the tumor but, rather, associate intimately with T cells, myeloid cells, and other immune cells inside TLSs that closely resemble LNs (6), it is possible that antibodies produced by plasma cells primed in tdLNs can exert similar effects. Importantly, B cells can present cognate tumor-derived antigens to CD4^+^ T helper cells and cytotoxic CD8^+^ T cells. In patients with colorectal cancer, carcinoembryonic antigen (CEA)-specific, CD21^low^ CD86^+^ B cells in tdLNs were able to induce IFN-γ secretion by autologous CD3^+^ T cells *in vitro* in the presence of a CEA peptide pool (84). Their roles as APCs make B cells potential targets for immunotherapies, as intra-tumoral injection of TLR9 agonist induced a body-wide immune response that required antigen presentation by B cells to naive T cells in tdLNs (85).

### 4.2 Tumor remodels draining lymph nodes to induce local and systemic immune tolerance

It is now well-established that the primary tumor actively remodels its tdLNs before colonization, creating pre-metastatic niches (86). A key aspect of this process is the alteration of LN vasculature, including increased HEV density and a shift to flatter endothelial cell phenotypes before tumor cell arrival (87). In addition, changes in LECs, such as local lymphatic expansion, compromised integrity, and the upregulation of adhesion molecules, also mark the formation of pre-metastatic niches (7). Fibroblastic reticular cells (FRCs), the primary extracellular matrix (ECM) producers in LNs, are also critical targets. FRCs mediate ECM remodeling, particularly by producing laminin α4 (88). *In vivo* models have shown a more proliferative, fibrotic FRC phenotype in pre-metastatic tdLNs, leading to fibrosis and restricted trafficking of lymphocytes and DCs (89). Other FRC alterations include downregulation of IL-7 and CCL21 (7) and suppression of FRC contractility, which lead to the relaxation of the FRC network (90). Notably, immunological changes were also shown to precede metastatic progression, as summarized in **Table 1**.

**Table 1:** Immunological alterations in lymph node pre-metastatic niches.

Cell type	Alteration	Tumor type(s)
Various types	Copy number aberrations and ectopic *KLK3* expression in immune cells, leading to micrometastases (91)	Prostate cancer
	Production of S1PR1/STAT3-activating factors by various cells, which enables myeloid cell colonization and consequent metastasis (92)	Melanoma and bladder cancer
	Increased activity of MDSCs (93)	Breast cancer
T cell	Lower levels of Th1 response (94)	Breast cancer
	Increased Treg activity and general anergy of T cells (93)	Breast cancer
B cell	B cell recruitment and proliferation; production of antibodies targeting HSPA4/ITGB5 that can activate Src/NF-κB signaling within tumor cells, ultimately supporting metastasis (8)	Breast cancer
DC	Decreased maturation and impaired activation (94)	Breast cancer
	Induction of Treg recruitment via COX-2/EP3/SDF-1 production (95)	Lewis lung carcinoma
	Accumulation of TAMs, resulting in increased VEGF and MMP production and IL-10 release (96)	Gastric cancer
	Increased PD-L1 expression on macrophages (97)	Non-small-cell lung cancer
Fibroblasts	IL-8 production by CAFs induces CD8^+^ T cells to upregulate PD-1 (92)	Gastric cancer
Neutrophils	Expansion and polarization of neutrophils in a granulocyte colony-stimulating factor-dependent manner, suppressing CD8^+^ T cells activity and promoting metastasis (98)	Spontaneous breast cancer
	Increased deposition of NETs, which promote LN metastases (99)	Melanoma and lung adenosquamous carcinoma

CAF, cancer-associated fibroblasts; DC, dendritic cell; MDSC, myeloid-derived suppressor cell; MMP, matrix metalloprotein; NET, neutrophil extracellular trap; PD-1, programmed death 1; TAM, tumor-associated macrophages; Th1, T helper type 1; Treg, regulatory T cell; VEGF, vascular endothelial growth factor.

Notably, whether or not a tdLN is colonized by tumor cells, immune cells inside the tdLN are under heavy suppression. Effector cells decrease in number and exhibit more immature or anergic phenotypes, and immune suppressive cells undergo expansion. Notably, mounting evidence suggests that tdLNs are progressively suppressed with disease progression, as cell populations in tdLNs with metastatic lesions exhibit heavier patterns of immune suppression. This progressive, tumor-specific immune tolerance has been observed in various LN-resident cell types, as discussed below.

#### 4.2.1 Immunosuppression in CD8^+^ T cells

As noted earlier, stem-like CD8^+^ T cells could develop into transitory effector cells before becoming terminally exhausted. Using prostate and kidney tumor models, a study showed that all activated CD8^+^ T cells in non-metastatic LNs were phenotypically stem-like (5). However, single-cell RNA-seq performed on tdLNs from melanoma patients showed increased exhaustion profiles in CD8^+^ T cells and a decrease in stem-like CD8^+^ T cells in metastatic LNs. The immunotolerant environment in metastatic tdLNs was further indicated by reduced CD8^+^ CD69^+^ and CD4^+^ CD69^+^ activated T cells, high PD-1 and CTLA-4 expression, and increased proportions of CD8^+^ CD57^+^ CD27^+^ PD-1^+^ effector cells (100). In addition, single-cell RNA-seq revealed that during LN metastasis, exhausted CD8^+^ T cells with high CXCL13 expression strongly interacted with tumor cells. This promoted tumor cells to acquire more aggressive phenotypes of extranodal expansion, which is associated with the poorest outcomes in patients with head and neck squamous cell carcinoma (101). Taken together, these studies demonstrate that the presence of tumor cells in the tdLN correlates with more severely suppressed CD8^+^ T cell responses.

In line with this, robust T cell responses to ICB seen in non-metastatic LNs are impaired in metastatic nodes. For HNSCC patients, Tpex were found to colocalize with DCs, regulatory T cells (Tregs), and CD4^+^ T cells exhibiting more immunosuppressive properties in metastatic vs. non-metastatic tdLNs. As a result, the expansion and activation of Tpex cell populations are suppressed in metastatic LNs compared to uninvolved LNs in response to immunotherapy (102).

#### 4.2.2 Immunosuppression in CD4^+^ T cells

CD4^+^ T cells also experience immunosuppression in tdLNs. Subsets of intratumoral CD4^+^ T cells were shown to be effectors, which exhibit anti-tumoral abilities. Th cells could control tumors by releasing cytokines like tumor necrosis factor (TNF) and IFNγ (103). A subset of cytotoxic CD4^+^ T cells was shown to kill cancer cells in an antigen-specific manner (104). However, using a spontaneous lung adenocarcinoma model, a study showed that naive tumor-specific CD4^+^ T cells activated and underwent proliferation in the tdLN do not differentiate into effectors, as they either become Tregs or enter a state of anergy from the early stage of tumor development. Moreover, these anergic cells do not accumulate in tumors (105). In addition, it is well established that Tregs enhance both primary tumor growth and the formation of distant metastasis in many tumors (106, 107). Expansion and increased suppressive profiles of Tregs following LN metastasis indicate progressive immunosuppression. Single-cell profiling of human breast cancer metastatic LNs (108) and an orthotopic murine model of breast cancer with spontaneous LN metastasis (109) both found expansion of LN Tregs upon LN metastasis. Moreover, Tregs isolated from metastatic LNs were more proliferative and expressed higher costimulatory markers PD-1 and CTLA-4 (108, 109).

#### 4.2.3 Immunosuppression in dendritic cells

It is also evident that DCs are suppressed in tdLNs. LN rDCs were critical for activating CD8^+^ T cells (110). However, LN resident DCs in the context of tumor exhibited suboptimal priming abilities compared with those in viral infection (111). Flow cytometric profiling of DCs from tdLNs also revealed impaired maturation in melanoma (112) and breast cancer patients (93). Progressive suppression of DCs was also notable. While intratumoral stem-like CD8^+^ T cells acquire dysfunctional features and decrease in number as tumors progress, their frequency in the tdLN remains stable. This reservoir of stem-like CD8^+^ T cells is maintained by cDC1s in tdLNs, which were shown to decrease in number with tumor progression (113). In addition, both migratory DCs (mDCs) and rDCs are polarized toward a cDC2 type in metastatic LNs, which expresses higher levels of PD-L1 compared with cDC1s (107).

#### 4.2.4 Immunosuppression in B cells

Although less well-characterized than adaptive responses, recent evidence indicates suppressed humoral immunity inside tdLNs. Unsupervised graph clustering found a cluster of TIM-1^+^ B cells that poorly infiltrated the tumor but preferentially aggregated within the tdLN in melanoma. These cells express high levels of various co-inhibitory and immunoregulatory molecules that are usually detected on T cells, and their selective deletion substantially enhanced the type 1 IFN response in B cells, enhanced effector T cell responses, and inhibited tumor growth (114). High accumulation of IL-10-producing regulatory B cells and plasmablasts was also found within tdLNs (115). Progressive suppression of humoral immunity is also observed in tdLNs. In mice with LN metastasis, T to B cell ratio reductions were observed in involved versus uninvolved nodes (107). In addition, LNs identified as non-metastatic by pathological examination had increased B cells of regulatory phenotype in node-positive breast cancer patients compared with patients without LN involvement (116).

#### 4.2.5 Immunosuppression in myeloid cells

Myeloid cells inside tdLNs, such as macrophages and neutrophils, are also immunosuppressed. In metastatic LNs, macrophages increase fraction and total number and exhibit elevated PD-L1 expression (107). Single-cell profiling revealed increased infiltration of fibroblasts and macrophages with tumor progression. These cells interacted with each other or with tumor cells to shape a desmoplastic microenvironment and reprogram malignant cells to promote tumor progression (101). In mice with LN metastases, neutrophils exhibit gene expression profiles associated with immature or immunosuppressive phenotypes (107).

#### 4.2.6 Immunosuppression in lymphatic endothelial cells

LECs in tdLNs have recently been recognized as direct regulators of tumor immune suppression. Loss of VE-cadherin expression in tdLN LECs increases lymphatic permeability, which was associated with increased visceral metastasis in melanoma patients (117). In the steady state, presentation of peptides derived from peripheral tissue self-antigens by LN LECs to CD8^+^ T cells contributes to the establishment of peripheral self-tolerance, and this mechanism is leveraged by tumor cells. A study observed uptake of melanoma extracellular vesicles (EVs) by LN LECs, which transfers tumor antigens to tolerogenic LN LECs, impairing CD8^+^ T-cell responses in those LNs (118). Another study showed that LECs restrain tumor-specific immunity via PD-L1 expression, with lymphatic PD-L1 deficiency resulting in consistent expansion of tumor-specific CD8^+^ T cells in tdLNs (119).

#### 4.2.7 Immunosuppression in fibroblastic reticular cells

FRCs residing in tdLNs also exhibit immunosuppressed profiles. Pathway analysis of deregulated genes in tdLNs suggests that FRCs undergo a metabolic shift toward oxidative phosphorylation, which is accompanied by a decrease in cytokine and chemokine responses (120). In metastatic LNs, researchers identified four cancer-associated fibroblast (CAF) subpopulations. Among these, the two most abundant CAF subsets are associated with cancer cell invasion. One is shown to promote cancer cell migration and triggers epithelial-to-mesenchymal transition, and the other facilitates cancer cell invasion and is positively associated with distant metastases (121).

## 5 Neural regulation of immune cell activities in the tumor microenvironment

Neuropeptides and neurotransmitters from sensory and sympathetic neurons, including NMU, CGRP, VIP, SP, and NE, were shown to control immune cell activities, as summarized in **Table 2**. Notably, neural regulation of immune cell activities is extensively studied in the TME, dependent and independent of neuropeptides and neurotransmitters.

**Table 2:** Immune regulation by neurotransmitters.

Neurotransmitter /neuropeptide	Cellular origins	Receptors	Signaling	Immune regulation
NMU	CNS of rat, mouse, and human; GIT of rats, pigs, and humans (submucosal and myenteric plexuses) (122); gut and ventromedial hypothalamus of rats (123); cholinergic neurons in GIT and lung of mice (124); thoracic DRG neurons of mice (125)	NMUR1 and NMUR2 in humans	Ca^2+^-calcineurin-NAFT and MAPK in mice (124)	Promote mast cell-mediated inflammation in mice (126) and humans (127)
				Promote ILC2 activation, induce protective type 2 responses (124) and allergic lung inflammation (125), all in mice
				Activate eosinophils and promote mucosal immunity in mice (25)
CGRP	CNS (brain and spinal cord) and TG of rat (128); NaV1.8^+^ nociceptors in meninge (129), skin (130), lung (131), LN (30), spleen (22), and colon (132) in mice; CD4^+^T cells in humans (133)	RAMP1, RAMP2, and RAMP3 in mice and humans (134)	PLCβ-Ca^2+^-PKC in mice and humans; AC-cAMP-PKA in mice, rats, and humans (135)	Suppress the recruitment of monocytes and neutrophils in S. aureus infection in mice (16)
				Suppress neutrophil and T cell responses in lung infections (131)
				Act on B cells to promote germinal center responses and humoral immunity (22)
				Suppress ILC2 function, constrain allergic airway inflammation (24) and worm expulsion (136)
				Trigger protective innate type 17 immunity (137)
				Produce memory Th2 cells and induce conjunctival itch (133)
				Attract DCs and enhance DCs’ inflammatory responses in mice (138)
				Impair efferocytosis and increased endometrial cell growth in macrophages in mice (139)
VIP	Brain interneurons in mice (140); enteric nerves in humans (141) and mice (142); sensory neurons in mice (143); sensory fibers in both central (thymus) and peripheral (spleen, lymph nodes, and mucosal-associated lymphoid tissue) lymphoid organs (144)	VPAC1, VPAC2, and PAC1 in humans	AC-cAMP-PKA in mice (145)	Inhibit production of IL-22 by ILC3 in mice (142).
				VIP-R antagonist improves T cell recruitment and activation in tumor-bearing mice (146).
				Dampen Th1 responses by inhibiting effector T cells and boosting regulatory T cells, relieving ICB-induced pneumonitis in humans (147)
				VIP^+^ sensory neuron ablation reduces B cell numbers, IgG release, and neutrophil stimulation in mice (143).
				Reduce proinflammatory serum cytokines IL-6 and IL-12 (148).
SP	TRPV1^+^ sensory neurons in mice (149); nasal epithelial cells in mice and humans (150)	NK1, NK2, and NK3 in humans	AC-cAMP-PKA in mice (151)	Induced DC migration to the LN, where they initiate Th2 differentiation in mice (149)
				Induce mast cell degranulation in mice (152)
				Mediate plasma extravasation, neutrophil recruitment, and diapedesis in mice (153)
NE	Adrenal medulla and postganglionic sympathetic neurons in humans and mice (154)	α1-, α2-, and β-AR in humans	AC-cAMP-PKA in mice and humans (155)	Limit immune cell extravasation in mice (156)
				Attenuate the innate antiviral response in mice (155).
				Attenuated noradrenergic input induces less immunosuppressive MDSCs in mice (157)
				Induce proinflammatory changes in monocytes in humans (158)
				Triggers myeloid progenitor proliferation and differentiation in mice (159)
				Inhibit CD8^+^ and CD4^+^ T cell locomotion, impair T cell responses to infections and tumors in mice (35)

AC, adenylyl cyclase; AR, adrenergic receptor; cAMP, cyclic adenosine monophosphate; CGRP, calcitonin gene-related peptide; CNS, central nervous system; DC, dendritic cell; DRG, dorsal root ganglia; GIT, gastrointestinal tract; ICB, immune checkpoint blockade; IL, interleukin; ILC, innate lymphoid cell; LN, lymph node; MAPK, mitogen-activated protein kinase; MDSC, myeloid-derived suppressor cell; NE, norepinephrine; NFAT, nuclear factor of activated T cell; NK1/2/3, neurokinin 1/2/3; NMU, neuromedin U; NMUR1/2, NMU receptor 1/2; PAC1, procaspase activating compound 1; PLC, phospholipase C; PKA, protein kinase A; PKC, protein kinase C; RAMP1/2/3, receptor-activity-modifying protein 1/2/3; S.aureus, Staphylococcus aureus; SP, substance P; Th, helper T cell; TRPV1, transient receptor potential vanilloid subfamily type 1; VIP, vasoactive intestinal peptide; VIP-R, VIP receptor; VPAC1/2, vasoactive intestinal peptide type 1/2.

Nerves can promote tumor immune evasion independent of neurotransmitters. In cutaneous squamous cell carcinoma, cancer cells induce nerve damage by degrading the myelin sheath, activating immune-suppressive pathways involving M2 macrophages and Tregs, ultimately diminishing the efficacy of anti-PD-1 therapy (160). Further, Schwann cells (SCs) drive CAFs toward a more malignant inflammatory phenotype via IL-1α signaling (161). In addition, in prostate cancer, tumor-infiltrating nerves express high levels of PD-L1, which correlates with reduced CD8^+^ T-cell presence and increased recurrence rates (162).

Regulation of immune cells in the TME by autonomic nerves has been extensively studied. Vagally modulated memory T cells release TFF2 to suppress the expansion of myeloid-derived suppressor cells (MDSCs) (163). In a mouse model of breast cancer, both stress-induced and pharmacologic β-adrenergic activation can induce macrophage infiltration into tumor parenchyma and differentiation into the immunosuppressive M2 phenotype. This resulted in increased expression of tumor growth factor (TGF)-β, vascular endothelial growth factor (VEGF), and matrix metalloprotein 9 (MMP-9) that enhanced angiogenesis and metastasis (164). As monotherapies, α2-adrenergic receptor agonists were found to enhance the ability of macrophages to activate T cells and demonstrated robust anti-tumor activity in multiple immunocompetent mouse models, including in models of ICB-resistant cancer and mice transplanted with human cancer cell xenografts and reconstituted with human lymphocytes (165). In various human and mouse tumors, exhausted CD8^+^ T cells upregulate the *adrenoceptor beta 1 (ADRB1)* gene, which programs β-adrenergic receptors, and are often distributed near sympathetic nerves. The exhaustion state can be induced when ADRB1-expressing CD8^+^ T cells are exposed to catecholamines. Combining β-blockers with ICB enhanced CD8^+^ T cell responses in ICB-resistant pancreatic cancer mouse model and melanoma treatment (166). In colorectal cancer, NE induces *adrenoceptor beta 2* (*ADRB2)*-dependent nerve growth factor (NGF) secretion from CAFs, which in turn increases intra-tumor sympathetic innervation and NE accumulation, leading to tumor cell growth and worse prognosis (167). Sciatic nerve stimulation enhances natural killer (NK) cell cytotoxicity through dopamine signaling in triple-negative breast cancer, improving the efficacy of ICB (168).

Sensory regulation of immune activity was also extensively studied. B16F10 melanoma cells induce nociceptor neurons to secrete CGRP, which induces exhaustion in receptor activity-modifying protein 1 (RAMP1)-expressing CD8^+^ T cells (169). Single-cell RNA-seq revealed that RAMP1-expressing CD8^+^ T cells exhibited greater exhaustion in melanoma, and the heightened expression of RAMP1 in these CD8^+^ T cells was linked to a diminished responsiveness to ICB (170). In addition, CGRP acts via RAMP1 on neutrophils, monocytes, and macrophages to inhibit recruitment, accelerate death, enhance efferocytosis, and polarize macrophages towards a pro-repair phenotype, potentially promoting tumor progression (130). VIP was observed at elevated plasma levels in patients with pancreatic ductal adenocarcinoma. VIP receptor (VIPR) was more prevalent in activated T cells. Inhibition of VIPR signaling boosted anti-tumor immunity, especially when combined with anti-PD1 treatment (146). Expression of CGRP in medullary thyroid cancer is associated with abnormal development of DCs characterized by activation of cAMP-related pathways and high levels of Kruppel Like Factor 2, and impaired activity of tumor-infiltrating T cells (171).

## 6 Tumor-nerve interactions

**Figure 3:**
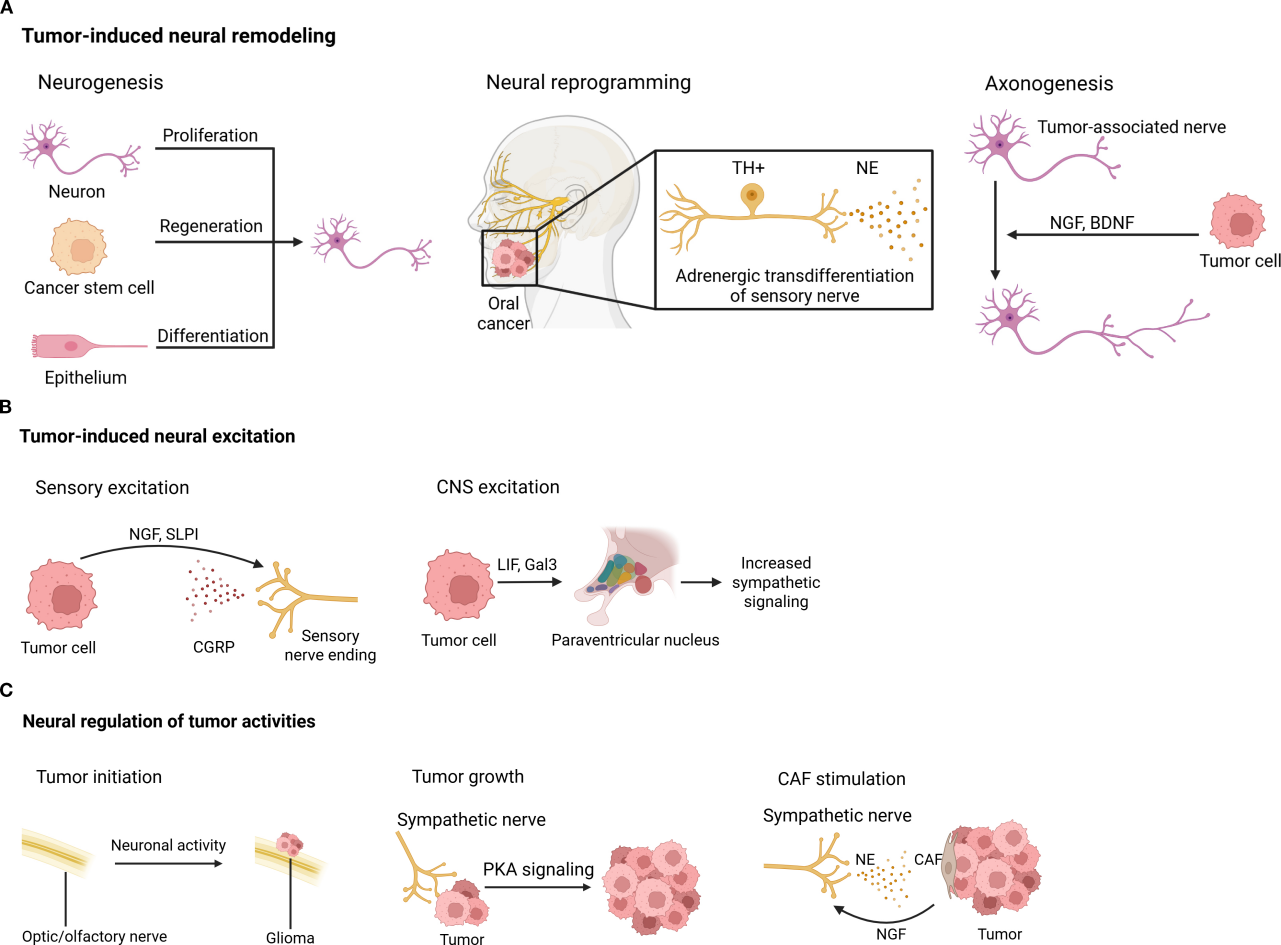
Tumor-induced neural remodeling. **(A)** Neural remodeling, including neurogenesis, neural reprogramming, and axonogenesis, happens in the tumor context. **(B)** Tumors can induce neural excitation in both the CNS and the periphery. **(C)** Conversely, neural signals control tumor activities directly via neurotransmitters/neuropeptides and indirectly by influencing other cells inside the TME. BDNF, brain-derived neurotrophic factor; CAF, cancer-associated fibroblast; CGRP, calcitonin gene-related peptide; CNS, central nervous system; Gal3, galectin-3; LIF, leukemia inhibitory factor; NE, norepinephrine; NGF, nerve growth factor; PKA, protein kinase A; SLPI, secreting secretory leukocyte protease inhibitor; TH, tyrosine hydroxylase.

Apart from interactions between nerves and immune cells, active tumor-nerve interplays have been documented in the TME. Cancer remodels the nervous system and induces neural excitation, facilitating oncogenesis, tumor growth, and metastatic spread (**Figure 3**).

### 6.1 Neural remodeling in the tumor context

Nerves directly innervating or in proximity to the TME undergo various modifications, including neurogenesis, neural reprogramming, and tumor-associated axonogenesis, as detailed below.

Neurogenesis, which is characterized by an increased number of neurons, can occur in tumor innervation. Neural progenitors originating from the CNS infiltrate prostate tumors and metastases, where they initiate neurogenesis and promote tumor growth and metastasis (172). In olfactory neuroblastoma, the olfactory epithelium undergoes neuronal regeneration from basal stem cells (173). Additionally, cancer stem cells derived from patients’ gastric and colorectal carcinomas have been shown to differentiate into TH-producing sympathetic neurons and vesicular Ach transporter-producing parasympathetic neurons. These neurons communicate reciprocally with cancer cells within xenografts, facilitating tumor growth (174).

Neural reprogramming, or the transformation between nerve types, has also been observed in tumor settings. Transcriptome analysis of cancer-associated trigeminal sensory neurons in mouse models of oral cancer revealed an adrenergic differentiation signature. Loss of TP53 leads to adrenergic transdifferentiation of tumor-associated sensory nerves via the downregulation of miR-34a. This reprogramming plays a crucial role in tumor progression, as tumor growth is inhibited by sensory denervation or pharmacological blockade of adrenergic receptors, but not by chemical sympathectomy of pre-existing adrenergic nerves (175).

Tumor-associated axonogenesis, involving nerve enlargement or increased nerve density, is one of the most extensively studied neural modifications in cancer. Tumor cells can promote nerve growth through the secretion of chemical factors. For example, DU-145 prostate cancer cells express semaphorin 4F, which induces neurite sprouting and elongation (176). NGF overexpression within the gastric epithelium expanded enteric nerves and promoted carcinogenesis (177). Brain-derived neurotrophic factor (BDNF), induced by noradrenergic signaling, stimulates axonogenesis through Trk receptors in pancreatic (178), ovarian (179), and colon cancers (179).

### 6.2 Tumor-induced neural excitation

Tumor-derived factors have been shown to induce neural excitation. Melanoma cells interact with nociceptor neurons by secreting secretory leukocyte protease inhibitor (SLPI), a small peptide that promotes neurite outgrowth, enhances responsiveness to noxious ligands, and stimulates the release of the neuropeptide CGRP (169). Similarly, breast cancer cells have been shown to trigger spontaneous calcium activity in sensory neurons, though the underlying mechanism remains unclear (28). In the low-glucose environments of oral mucosal carcinomas and melanoma, cancer cells respond to nutrient starvation by secreting NGF, which stimulates nociceptive neurons to release CGRP. Elevated CGRP levels subsequently promote tumor progression (180). Interestingly, recent studies suggest that blood-borne tumor-derived leukemia inhibitory factor (LIF) and galectin-3 (Gal3) may excite the paraventricular nucleus of the hypothalamus, leading to increased sympathetic signaling (181).

### 6.3 Neural regulation of tumor activities

Conversely, neural signals have been shown to control tumor activities in both CNS and periphery tumors. Neuronal activity driven by neurofibromatosis 1 (NF1) mutation drives the initiation of optic glioma (182). Insulin-like growth factor 1 (IGF1), a neuronal activity-regulated paracrine signaling molecule, mediates olfactory sensory experience-dependent initiation of olfactory bulb high-grade glioma (183). Moreover, neuronal activity promotes glioma growth through neuroligin-3 secretion (184). Beyond the CNS, many studies have also demonstrated neural regulation of tumor pathogenesis. A recent study showed that sympathetic nerve fibers are enriched in mouse small cell lung cancer (SCLC) and promote tumor growth by activating protein kinase A (PKA) signaling (185). Perineural invasion is commonly seen in pancreatic ductal adenocarcinoma (PDAC), and has been shown to trigger proliferation and migration of pancreatic cancer cells and induce angiogenesis (186). A recent study further validated Neural regulation in PDAC by characterizing single sympathetic and sensory neurons (187). Additionally, adrenergic stimulation accelerates colorectal cancer growth through activation of Yes-associated protein (YAP) (167), and 5-hydroxytryptamine produced by enteric serotonergic neurons initiates colorectal cancer stem cells (188). Furthermore, hyperexcitation of DRG sensory neurons can drive the development of peripheral nerve sheath tumors (189). Nerves also regulate tumors indirectly via non-tumor cells in the TME. In colorectal cancer, NE induces *ADRB2*-dependent NGF secretion from CAFs, which directly enhances colorectal cancer cell growth via the phosphatidylinositol-3-kinase/AKT pathway (167).

## 7 Potential tumor-neuro-immune interactions in tumor-draining lymph nodes

Tumor cells, nerves, and immune cells coexist inside tdLNs, where anti-tumor immunity is initiated and compromised. Although validation is needed, accumulating evidence suggests that tumor-neuro-immune interactions may take place in tdLNs.

Tumor-derived factors may cause remodeling and/or stimulation of LN-innervating fibers. Sensory innervation often increases at sites of inflammation (190), and lipopolysaccharides (LPS) challenge leads to a consistent two-fold increase in sensory fiber content in draining LNs (30). However, this increase is independent of direct recognition of TLR ligands by sensory neurons (30), suggesting that LN expansion may be a shared feature of inflammatory responses of various origins. During tumor progression, tumor cells and immune cells release a cocktail of factors, including cytokines, histamine, lipids, and growth factors (191), whose receptors are located on nociceptive sensory neurons (192). Many of these factors have been found in tdLNs, as summarized in **Table 3**, suggesting potential nociceptor activation.

**Table 3:** Nociceptor-activating immune molecules inside tdLNs.

Immune molecule	Cellular origin	Tumor types	Receptors in DRG neurons	Neural stimulatory effects
GM-CSF (1)	Tumor cells	Fibrosarcoma in mice	Csf2ra, Csf2rb	Increase mechanical and thermal hypersensitivity (193)
IL-3 (1)	Tumor cells	Fibrosarcoma in mice	IL-3ra, Csf2rb	Induce neuroprotection (192)
IL-10 (194)	NA	Melanoma in humans	NA	Downregulate mechanical sensitivity (192)
IL-4 (71)	Tfh	Colorectal cancer in mice	IL-4ra	Activate sensory neurons (195)
IL-33 (196)	MSMs	Melanoma in mice and humans	NA	Mediate articular and cutaneous hypernociception (192).
CCL21	FRCs (7)	Melanoma in mice	NA	Increase intracellular Ca^2+^ (192)
IL-1	Tumor cells (90)	Melanoma in mice	IL1r1, IL1r2	Augment neuron excitability (192)
IFNγ	CD8^+^ T cells (79)	Melanoma in mice	NA	Induce mechanical hypersensitivity (192)

Csf2ra/b, colony-stimulating factor 2 receptor a/b; GM-CSF, granulocyte-macrophage colony-stimulating factor; IFNγ, interferon-gamma; IL, interleukin; IL1r1/2, IL-1 receptor 1/2; IL-3/4ra, IL-3/4 receptor a; MSM, medullary sinus macrophage; NA, not available; Tfh, follicular helper T cell.

Moreover, tumor-derived EVs (tdEVs) are now recognized as mediators of LN remodeling (118), immunosuppression inside LNs (118), and LN metastasis (99, 197). TdEVs have been shown to induce neurite outgrowth and neural excitation. They can induce the expression of various cytokines and chemokines by immune and nonimmune cells (198). Moreover, tdEVs were shown to recruit nerves by delivering neurotrophic factors. Genetically aberrant, p53-knockout or -mutant oral squamous cell carcinoma cells release tdEVs that promote axonogenesis in DRG (175). In cervical cancer, sensory axonogenesis is enhanced by exosome-packaged EphrinB1, an axonal guidance molecule (199). In pancreatic cancer mouse models, extracellular vesicle-packaged PNI-associated transcript (PIAT) from CAFs mediates m5C modification, driving nerve infiltration into tumors (200). Since tdLNs are densely innervated and enriched with tdEVs, the observed alterations in tdLNs may result from tdEV-induced neural activities.

Beyond chemical stimuli, physical factors may also contribute to sensory nerve activation within tdLNs. Elevated solid stress has been shown in tdLNs of multiple tumor types in mouse models as well as human patients (201). Notably, within an LN, SCS is the region with the highest pressure (202), and SCS thickness increases most significantly during inflammation or tumor drainage (203). SCS is the first site of LN metastasis (204), and dilation of the SCS—initiating at its junctions with afferent lymphatic vessels—precedes the arrival of tumor cells (204). Given the dense capsular/subcapsular sensory network in LNs, pressure-induced activation of sensory nerves may represent a mechanism underlying early immunosuppression in tdLNs. Interestingly, while nociceptors innervating LNs are mainly peptidergic (30), some studies have reported the presence of Pacinian corpuscles, mechanoreceptors associated with blood vessels, in the hilar region of LNs resected from cancer patients (205). Although their precise functions remain unclear, it is plausible that these mechanoreceptors could detect pressure changes in enlarged tdLNs.

## 8 Current perspectives on lymph node handling in cancer therapy and clinical implications

Extensive research has been conducted regarding LN handling in cancer therapy. To what extent tdLNs should be removed, if at all, remains controversial (11, 206–208). Even when metastasis is already present in tdLNs, extensive LN dissection does not appear to confer additional survival benefits (209). In addition, studies are increasingly focused on leveraging LNs to combat tumors, both in traditional immunotherapies and novel LN-targeted treatments. In murine models, neoadjuvant ICB (ICB conducted before surgery) has demonstrated improved cure rates compared to adjuvant ICB (ICB conducted after surgery) (12). In line with this, neoadjuvant anti-PD-1 therapy significantly improves pathological complete response rates and event-free survival in patients with various malignancies (13). Moreover, radiation of LNs before ICB also disrupts responses to ICB in preclinical models (210). As noted earlier, Tpex cells are less responsive to ICB in involved LNs, and tdLNs experience progressive immunosuppression with tumor progression. Therefore, it has been proposed that LN resection may be beneficial for late-stage cancers, while preserving tdLNs in earlier stages may improve outcomes, warranting further research (4, 10). Interestingly, a recent study showed that prophylactic lymphangiogenesis was associated with enhanced tumor eradication, which was associated with an increase in immunocompetent cells in the deep cervical LNs (211), linking tdLNs with tumor prevention.

Current research is further looking into the clinical values of tdLNs. TdLN-targeted therapies have yielded promising results, especially. In mouse models, targeted delivery of ICB to tdLNs enhances therapeutic efficacy (14, 77). In line with this, clinical trials using intradermal administration of immune adjuvants or ICB to enhance LN priming of anti-tumor responses have shown promising results (15, 212). As proposed in this review, tripartite tumor-neuro-immune crosstalk potentially exists inside tdLNs. Although the impact of nerve ablation or nerve growth promotion on tumor progression—both positive and negative—has been extensively investigated in animal models and clinical studies (26, 27), targeting tdLN-innervating fibers may be a promising new direction. Notably, simultaneous targeting of both sympathetic and sensory nerves would be necessary. Since tumor-associated sensory neurons can be reprogrammed toward an adrenergic phenotype (175), compensatory reprogramming of the remaining neurons may occur following selective ablation of one neural subtype.

## 9 Conclusions

In conclusion, we have highlighted the interactions between the nervous system, LNs, and malignancies. Recent progress in cancer neuroscience and oncoimmunology has shed light on the nerve-immune and tumor-nerve interplays almost exclusively within tumors (26, 27). In contrast, the roles of LNs in tumor immunity and cancer immunotherapy have long been less investigated. TdLNs have multifaceted roles in tumor immunology, including orchestration of anti-tumor immunity and local immunosuppression, pre-metastatic LN remodeling, and induction of systemic tumor-specific immune tolerance (87, 88, 107, 118). Recent studies revealed that LNs are densely innervated by sensory and sympathetic nerve fibers (28, 30). In addition, immune activities, especially those within LNs and those associated with a tumor context, are regulated by neurotransmitters including NMU, CGRP, VIP, SP, and NE (22, 24). Moreover, intricate interplay exists between tumors and nerves, including neural remodeling, tumor-induced neural activation, and neural regulation of tumor activities (165, 185, 200). Collectively, tripartite tumor-neuro-immune interactions may exist within tdLNs, potentially associated with anti-tumor immunity and tumor immune evasion. Further studies are needed to validate this hypothesis. This should involve primary evidence, such as transcriptomic mapping to show spatial co-localization of tumor cells, neural terminals, and immune cells, or functional validation of paracrine circuits through ex vivo tdLN co-culture systems. Ultimately, studies exploring the relationships between LN innervation, anti-tumor immunity, and tumor immune evasion can facilitate optimal LN handling in cancer therapy and inspire better therapeutic strategies.

## References

[1] Cruz de Casas P Knöpper K Dey Sarkar R Kastenmüller W. Same yet different - how lymph node heterogeneity affects immune responses. Nat Rev Immunol. (2024) 24:358–74. doi: 10.1038/s41577-023-00965-8, PMID: 38097778

[2] Koning JJ Mebius RE. Interdependence of stromal and immune cells for lymph node function. Trends In Immunol. (2012) 33:264–70. doi: 10.1016/j.it.2011.10.006, PMID: 22153930

[3] Swanton C Bernard E Abbosh C André F Auwerx J Balmain A. Embracing cancer complexity: Hallmarks of systemic disease. Cell. (2024) 187:1589–616. doi: 10.1016/j.cell.2024.02.009, PMID: 38552609 PMC12077170

[4] Reticker-Flynn NE Engleman EG. Lymph nodes: at the intersection of cancer treatment and progression. Trends Cell Biol. (2023) 33:1021–34. doi: 10.1016/j.tcb.2023.04.001, PMID: 37149414 PMC10624650

[5] Prokhnevska N Cardenas MA Valanparambil RM Sobierajska E Barwick BG Jansen C. CD8+ T cell activation in cancer comprises an initial activation phase in lymph nodes followed by effector differentiation within the tumor. Immunity. (2023) 56. doi: 10.1016/j.immuni.2022.12.002, PMID: 36580918 PMC10266440

[6] Laumont CM Banville AC Gilardi M Hollern DP Nelson BH. Tumour-infiltrating B cells: immunological mechanisms, clinical impact and therapeutic opportunities. Nat Rev Cancer. (2022) 22:414–30. doi: 10.1038/s41568-022-00466-1, PMID: 35393541 PMC9678336

[7] Riedel A Shorthouse D Haas L Hall BA Shields J. Tumor-induced stromal reprogramming drives lymph node transformation. Nat Immunol. (2016) 17:1118–27. doi: 10.1038/ni.3492, PMID: 27400148 PMC4994871

[8] Gu Y Liu Y Fu L Zhai L Zhu J Han Y. Tumor-educated B cells selectively promote breast cancer lymph node metastasis by HSPA4-targeting IgG. Nat Med. (2019) 25:312–22. doi: 10.1038/s41591-018-0309-y, PMID: 30643287

[9] Delclaux I Ventre KS Jones D Lund AW. The tumor-draining lymph node as a reservoir for systemic immune surveillance. Trends In Cancer. (2024) 10:28–37. doi: 10.1016/j.trecan.2023.09.006, PMID: 37863720 PMC10843049

[10] Saddawi-Konefka R Schokrpur S Gutkind JS. Let it be: Preserving tumor-draining lymph nodes in the era of immuno-oncology. Cancer Cell. (2024) 42:930–3. doi: 10.1016/j.ccell.2024.05.015, PMID: 38861928

[11] de Boniface J Filtenborg Tvedskov T Rydén L Szulkin R Reimer T Kühn T. Omitting axillary dissection in breast cancer with sentinel-node metastases. N Engl J Med. (2024) 390:1163–75. doi: 10.1056/NEJMoa2313487, PMID: 38598571

[12] Liu J Blake SJ Yong MCR Harjunpää H Ngiow SF Takeda K. Improved efficacy of neoadjuvant compared to adjuvant immunotherapy to eradicate metastatic disease. Cancer Discov. (2016) 6:1382–99. doi: 10.1158/2159-8290.CD-16-0577, PMID: 27663893

[13] Sun J-M Shen L Shah MA Enzinger P Adenis A Doi T. Pembrolizumab plus chemotherapy versus chemotherapy alone for first-line treatment of advanced oesophageal cancer (KEYNOTE-590): a randomised, placebo-controlled, phase 3 study. Lancet. (2021) 398:759–71. doi: 10.1016/S0140-6736(21)01234-4, PMID: 34454674

[14] Francis DM Manspeaker MP Schudel A Sestito LF O’Melia MJ Kissick HT. Blockade of immune checkpoints in lymph nodes through locoregional delivery augments cancer immunotherapy. Sci Transl Med. (2020) 12. doi: 10.1126/scitranslmed.aay3575, PMID: 32998971 PMC8377700

[15] van Pul KM Notohardjo JCL Fransen MF Koster BD Stam AGM Chondronasiou D. Local delivery of low-dose anti-CTLA-4 to the melanoma lymphatic basin leads to systemic Treg reduction and effector T cell activation. Sci Immunol. (2022) 7:eabn8097. doi: 10.1126/sciimmunol.abn8097, PMID: 35857579

[16] Chiu IM Heesters BA Ghasemlou N Von Hehn CA Zhao F Tran J. Bacteria activate sensory neurons that modulate pain and inflammation. Nature. (2013) 501:52–7. doi: 10.1038/nature12479, PMID: 23965627 PMC3773968

[17] Zhu Y Meerschaert KA Galvan-Pena S Bin NR Yang D Basu H. A chemogenetic screen reveals that Trpv1-expressing neurons control regulatory T cells in the gut. Science. (2024) 385:eadk1679. doi: 10.1126/science.adk1679, PMID: 39088603 PMC11416019

[18] Katayama Y Battista M Kao WM Hidalgo A Peired AJ Thomas SA. Signals from the sympathetic nervous system regulate hematopoietic stem cell egress from bone marrow. Cell. (2006) 124:407–21. doi: 10.1016/j.cell.2005.10.041, PMID: 16439213

[19] Gao X Zhang D Xu C Li H Caron KM Frenette PS. Nociceptive nerves regulate haematopoietic stem cell mobilization. Nature. (2021) 589:591–6. doi: 10.1038/s41586-020-03057-y, PMID: 33361809 PMC7856173

[20] Maryanovich M Zahalka AH Pierce H Pinho S Nakahara F Asada N. Adrenergic nerve degeneration in bone marrow drives aging of the hematopoietic stem cell niche. Nat Med. (2018) 24:782–91. doi: 10.1038/s41591-018-0030-x, PMID: 29736022 PMC6095812

[21] Carpenter RS Lagou MK Karagiannis GS Maryanovich M. Neural regulation of the thymus: past, current, and future perspectives. Front Immunol. (2025) 16:1552979. doi: 10.3389/fimmu.2025.1552979, PMID: 40046055 PMC11880003

[22] Wu M Song G Li J Song Z Zhao B Liang L. Innervation of nociceptor neurons in the spleen promotes germinal center responses and humoral immunity. Cell. (2024) 187:2935–51.e19. doi: 10.1016/j.cell.2024.04.027, PMID: 38772371

[23] Zhu X Huang J-Y Dong W-Y Tang H-D Xu S Wu Q. Somatosensory cortex and central amygdala regulate neuropathic pain-mediated peripheral immune response via vagal projections to the spleen. Nat Neurosci. (2024) 27:471–83. doi: 10.1038/s41593-023-01561-8, PMID: 38291284

[24] Tamari M Del Bel KL Ver Heul AM Zamidar L Orimo K Hoshi M. Sensory neurons promote immune homeostasis in the lung. Cell. (2024) 187. doi: 10.1016/j.cell.2023.11.027, PMID: 38134932 PMC10811756

[25] Li Y Liu S Zhou K Wang Y Chen Y Hu W. Neuromedin U programs eosinophils to promote mucosal immunity of the small intestine. Science. (2023) 381:1189–96. doi: 10.1126/science.ade4177, PMID: 37708282

[26] Magnon C Hondermarck H. The neural addiction of cancer. Nat Rev Cancer. (2023) 23:317–34. doi: 10.1038/s41568-023-00556-8, PMID: 37041409

[27] Mancusi R Monje M. The neuroscience of cancer. Nature. (2023) 618:467–79. doi: 10.1038/s41586-023-05968-y, PMID: 37316719 PMC11146751

[28] Padmanaban V Keller I Seltzer ES Ostendorf BN Kerner Z Tavazoie SF. Neuronal substance P drives metastasis through an extracellular RNA-TLR7 axis. Nature. (2024) 633:207–15. doi: 10.1038/s41586-024-07767-5, PMID: 39112700 PMC11633843

[29] Cleypool CGJ Mackaaij C Lotgerink Bruinenberg D Schurink B Bleys R. Sympathetic nerve distribution in human lymph nodes. J Anat. (2021) 239:282–9. doi: 10.1111/joa.13422, PMID: 33677834 PMC8273593

[30] Huang S Ziegler CGK Austin J Mannoun N Vukovic M Ordovas-Montanes J. Lymph nodes are innervated by a unique population of sensory neurons with immunomodulatory potential. Cell. (2021) 184:441–59 e25. doi: 10.1016/j.cell.2020.11.028, PMID: 33333021 PMC9612289

[31] De Virgiliis F Oliva VM Kizil B Scheiermann C. Control of lymph node activity by direct local innervation. Trends Neurosci. (2022) 45:704–12. doi: 10.1016/j.tins.2022.06.006, PMID: 35820971

[32] Murray K Barboza M Rude KM Brust-Mascher I Reardon C. Functional circuitry of neuro-immune communication in the mesenteric lymph node and spleen. Brain Behav Immun. (2019) 82:214–23. doi: 10.1016/j.bbi.2019.08.188, PMID: 31445965 PMC6800652

[33] Felten DL Livnat S Felten SY Carlson SL Bellinger DL Yeh P. Sympathetic innervation of lymph nodes in mice. Brain Res Bull. (1984) 13:693–9. doi: 10.1016/0361-9230(84)90230-2, PMID: 6532515

[34] Hanes WM Olofsson PS Talbot S Tsaava T Ochani M Imperato GH. Neuronal circuits modulate antigen flow through lymph nodes. Bioelectron Med. (2016) 3:18–28. doi: 10.15424/bioelectronmed.2016.00001, PMID: 33145374 PMC7604943

[35] Devi S Alexandre YO Loi JK Gillis R Ghazanfari N Creed SJ. Adrenergic regulation of the vasculature impairs leukocyte interstitial migration and suppresses immune responses. Immunity. (2021) 54. doi: 10.1016/j.immuni.2021.03.025, PMID: 33915109

[36] Nakai A Hayano Y Furuta F Noda M Suzuki K. Control of lymphocyte egress from lymph nodes through β2-adrenergic receptors. J Exp Med. (2014) 211:2583–98. doi: 10.1084/jem.20141132, PMID: 25422496 PMC4267238

[37] Tracey KJ. Lymphocyte called home: β2-adreneric neurotransmission confines T cells to lymph nodes to suppress inflammation. J Exp Med. (2014) 211:2483–4. doi: 10.1084/jem.21113insight3, PMID: 25512581 PMC4267244

[38] Chen CS Weber J Holtkamp SJ Ince LM de Juan A Wang C. Loss of direct adrenergic innervation after peripheral nerve injury causes lymph node expansion through IFN-γ. J Exp Med. (2021) 218. doi: 10.1084/jem.20202377, PMID: 34086056 PMC8185988

[39] van de Pavert SA Mebius RE. New insights into the development of lymphoid tissues. Nat Rev Immunol. (2010) 10:664–74. doi: 10.1038/nri2832, PMID: 20706277

[40] Teillaud J-L Houel A Panouillot M Riffard C Dieu-Nosjean M-C. Tertiary lymphoid structures in anticancer immunity. Nat Rev Cancer. (2024) 24:629–46. doi: 10.1038/s41568-024-00728-0, PMID: 39117919

[41] Riffard C Letaïef L Azar S Casrouge A Brunet I Teillaud J-L. Absence of sympathetic innervation hampers the generation of tertiary lymphoid structures upon acute lung inflammation. Sci Rep. (2024) 14:11749. doi: 10.1038/s41598-024-62673-0, PMID: 38782985 PMC11116507

[42] Vats K Kruglov O Sahoo B Soman V Zhang J Shurin GV. Sensory nerves impede the formation of tertiary lymphoid structures and development of protective antimelanoma immune responses. Cancer Immunol Res. (2022) 10:1141–54. doi: 10.1158/2326-6066.CIR-22-0110, PMID: 35834791 PMC10314799

[43] Amisaki M Zebboudj A Yano H Zhang SL Payne G Chandra AK. IL-33-activated ILC2s induce tertiary lymphoid structures in pancreatic cancer. Nature. (2025) 638:1076–84. doi: 10.1038/s41586-024-08426-5, PMID: 39814891 PMC11864983

[44] Coley WB. The Treatment of Inoperable Sarcoma by Bacterial Toxins (the Mixed Toxins of the Streptococcus erysipelas and the Bacillus prodigiosus). Proc R Soc Med. (1910) 3:1–48. doi: 10.1177/003591571000301601, PMID: 19974799 PMC1961042

[45] Burnet M. Cancer; a biological approach. I. The processes of control. Br Med J. (1957) 1:779–86. doi: 10.1136/bmj.1.5022.779, PMID: 13404306 PMC1973174

[46] Carr I. Lymphatic metastasis. Cancer Metastasis Rev. (1983) 2:307–17. doi: 10.1007/BF00048483, PMID: 6367969

[47] Billingham RE Brent L Medawar PB. Actively acquired tolerance of foreign cells. Nature. (1953) 172:603–6. doi: 10.1038/172603a0, PMID: 13099277

[48] Naxerova K Reiter JG Brachtel E Lennerz JK van de Wetering M Rowan A. Origins of lymphatic and distant metastases in human colorectal cancer. Science. (2017) 357:55–60. doi: 10.1126/science.aai8515, PMID: 28684519 PMC5536201

[49] Reiter JG Hung W-T Lee IH Nagpal S Giunta P Degner S. Lymph node metastases develop through a wider evolutionary bottleneck than distant metastases. Nat Genet. (2020) 52:692–700. doi: 10.1038/s41588-020-0633-2, PMID: 32451459 PMC7343611

[50] Brown M Assen FP Leithner A Abe J Schachner H Asfour G. Lymph node blood vessels provide exit routes for metastatic tumor cell dissemination in mice. Science. (2018) 359:1408–+. doi: 10.1126/science.aal3662, PMID: 29567714

[51] Pereira ER Kedrin D Seano G Gautier O Meijer EFJ Jones D. Lymph node metastases can invade local blood vessels, exit the node, and colonize distant organs in mice. Science. (2018) 359:1403–7. doi: 10.1126/science.aal3622, PMID: 29567713 PMC6002772

[52] Mellman I Chen DS Powles T Turley SJ. The cancer-immunity cycle: Indication, genotype, and immunotype. Immunity. (2023) 56:2188–205. doi: 10.1016/j.immuni.2023.09.011, PMID: 37820582

[53] Guo M Liu MYR Brooks DG. Regulation and impact of tumor-specific CD4(+) T cells in cancer and immunotherapy. Trends Immunol. (2024) 45:303–13. doi: 10.1016/j.it.2024.02.005, PMID: 38508931

[54] Lehmann J Thelen M Kreer C Schran S García-Marquez MA Cisic I. Tertiary lymphoid structures in pancreatic cancer are structurally homologous, share gene expression patterns and B-cell clones with secondary lymphoid organs but show increased T-cell activation. Cancer Immunol Res. (2024) 13:323–36. doi: 10.1158/2326-6066.CIR-24-0299, PMID: 39661055

[55] Chu T Wu M Hoellbacher B de Almeida GP Wurmser C Berner J. Precursors of exhausted T cells are preemptively formed in acute infection. Nature. (2025) 640:782–92. doi: 10.1038/s41586-024-08451-4, PMID: 39778709 PMC12003159

[56] Fang Z Ding X Huang H Jiang H Jiang J Zheng X. Revolutionizing tumor immunotherapy: unleashing the power of progenitor exhausted T cells. Cancer Biol Med. (2024) 21:499–512. doi: 10.20892/j.issn.2095-3941.2024.0105, PMID: 38825813 PMC11208905

[57] Magen A Hamon P Fiaschi N Soong BY Park MD Mattiuz R. Intratumoral dendritic cell-CD4(+) T helper cell niches enable CD8(+) T cell differentiation following PD-1 blockade in hepatocellular carcinoma. Nat Med. (2023) 29:1389–99. doi: 10.1038/s41591-023-02345-0, PMID: 37322116 PMC11027932

[58] Burger ML Cruz AM Crossland GE Gaglia G Ritch CC Blatt SE. Antigen dominance hierarchies shape TCF1+ progenitor CD8 T cell phenotypes in tumors. Cell. (2021) 184. doi: 10.1016/j.cell.2021.08.020, PMID: 34534464 PMC8522630

[59] Duckworth BC Lafouresse F Wimmer VC Broomfield BJ Dalit L Alexandre YO. Effector and stem-like memory cell fates are imprinted in distinct lymph node niches directed by CXCR3 ligands. Nat Immunol. (2021) 22:434–48. doi: 10.1038/s41590-021-00878-5, PMID: 33649580

[60] Huang Q Wu X Wang Z Chen X Wang L Lu Y. The primordial differentiation of tumor-specific memory CD8(+) T cells as bona fide responders to PD-1/PD-L1 blockade in draining lymph nodes. Cell. (2022) 185:4049–66.e25. doi: 10.1016/j.cell.2022.09.020, PMID: 36208623

[61] Molodtsov AK Khatwani N Vella JL Lewis KA Zhao Y Han J. Resident memory CD8+ T cells in regional lymph nodes mediate immunity to metastatic melanoma. Immunity. (2021) 54. doi: 10.1016/j.immuni.2021.08.019, PMID: 34525340 PMC9015193

[62] Pai JA Hellmann MD Sauter JL Mattar M Rizvi H Woo HJ. Lineage tracing reveals clonal progenitors and long-term persistence of tumor-specific T cells during immune checkpoint blockade. Cancer Cell. (2023) 41. doi: 10.1016/j.ccell.2023.03.009, PMID: 37001526 PMC10563767

[63] Wijesinghe SKM Rausch L Gabriel SS Galletti G De Luca M Qin L. Lymph-node-derived stem-like but not tumor-tissue-resident CD8+ T cells fuel anticancer immunity. Nat Immunol. (2025) 26:1367–83. doi: 10.1038/s41590-025-02219-2, PMID: 40730900

[64] Manspeaker MP O’Melia MJ Thomas SN. Elicitation of stem-like CD8(+) T cell responses via lymph node-targeted chemoimmunotherapy evokes systemic tumor control. J Immunother Cancer. (2022) 10. doi: 10.1136/jitc-2022-005079, PMID: 36100312 PMC9472119

[65] Escobar G Tooley K Oliveras JP Huang L Cheng H Bookstaver ML. Tumor immunogenicity dictates reliance on TCF1 in CD8(+) T cells for response to immunotherapy. Cancer Cell. (2023) 41:1662–79.e7. doi: 10.1016/j.ccell.2023.08.001, PMID: 37625402 PMC10529353

[66] Ferris ST Durai V Wu R Theisen DJ Ward JP Bern MD. cDC1 prime and are licensed by CD4+ T cells to induce anti-tumour immunity. Nature. (2020) 584:624–9. doi: 10.1038/s41586-020-2611-3, PMID: 32788723 PMC7469755

[67] Yang Y Chen X Pan J Ning H Zhang Y Bo Y. Pan-cancer single-cell dissection reveals phenotypically distinct B cell subtypes. Cell. (2024) 187:4790–4811.e22. doi: 10.1016/j.cell.2024.06.038, PMID: 39047727

[68] Bos R Sherman LA. CD4+ T-cell help in the tumor milieu is required for recruitment and cytolytic function of CD8+ T lymphocytes. Cancer Res. (2010) 70:8368–77. doi: 10.1158/0008-5472.CAN-10-1322, PMID: 20940398 PMC2970736

[69] Guo W Tan J Wang L Egelston CA Simons DL Ochoa A. Tumor draining lymph nodes connected to cold triple-negative breast cancers are characterized by Th2-associated microenvironment. Nat Commun. (2024) 15:8592. doi: 10.1038/s41467-024-52577-y, PMID: 39366933 PMC11452381

[70] Franken A Bila M Mechels A Kint S Van Dessel J Pomella V. CD4+ T cell activation distinguishes response to anti-PD-L1+anti-CTLA4 therapy from anti-PD-L1 monotherapy. Immunity. (2024) 57:541–558.e7. doi: 10.1016/j.immuni.2024.02.007, PMID: 38442708

[71] Ruggiu M Guérin MV Corre B Bardou M Alonso R Russo E. Anti-PD-1 therapy triggers Tfh cell-dependent IL-4 release to boost CD8 T cell responses in tumor-draining lymph nodes. J Exp Med. (2024) 221:e20232104. doi: 10.1084/jem.20232104, PMID: 38417020 PMC10901238

[72] Zhivaki D Kennedy SN Park J Boriello F Devant P Cao A. Correction of age-associated defects in dendritic cells enables CD4+ T cells to eradicate tumors. Cell. (2024) 187:3888–3903.e18. doi: 10.1016/j.cell.2024.05.026, PMID: 38870946 PMC11283364

[73] Jhunjhunwala S Hammer C Delamarre L. Antigen presentation in cancer: insights into tumour immunogenicity and immune evasion. Nat Rev Cancer. (2021) 21:298–312. doi: 10.1038/s41568-021-00339-z, PMID: 33750922

[74] Ruhland MK Roberts EW Cai E Mujal AM Marchuk K Beppler C. Visualizing synaptic transfer of tumor antigens among dendritic cells. Cancer Cell. (2020) 37:786–799.e5. doi: 10.1016/j.ccell.2020.05.002, PMID: 32516589 PMC7671443

[75] Meiser P Knolle MA Hirschberger A de Almeida GP Bayerl F Lacher S. A distinct stimulatory cDC1 subpopulation amplifies CD8+ T cell responses in tumors for protective anti-cancer immunity. Cancer Cell. (2023) 41:1498–1515.e10. doi: 10.1016/j.ccell.2023.06.008, PMID: 37451271

[76] Heras-Murillo I Adán-Barrientos I Galán M Wculek SK Sancho D. Dendritic cells as orchestrators of anticancer immunity and immunotherapy. Nat Rev Clin Oncol. (2024) 21:257–77. doi: 10.1038/s41571-024-00859-1, PMID: 38326563

[77] Dammeijer F van Gulijk M Mulder EE Lukkes M Klaase L van den Bosch T. The PD-1/PD-L1-checkpoint restrains T cell immunity in tumor-draining lymph nodes. Cancer Cell. (2020) 38:685–700.e8. doi: 10.1016/j.ccell.2020.09.001, PMID: 33007259

[78] He M Roussak K Ma F Borcherding N Garin V White M. CD5 expression by dendritic cells directs T cell immunity and sustains immunotherapy responses. Science. (2023) 379:eabg2752. doi: 10.1126/science.abg2752, PMID: 36795805 PMC10424698

[79] Shapir Itai Y Barboy O Salomon R Bercovich A Xie K Winter E. Bispecific dendritic-T cell engager potentiates anti-tumor immunity. Cell. (2024) 187:375–89.e18. doi: 10.1016/j.cell.2023.12.011, PMID: 38242085

[80] Gardner A de Mingo Pulido Á Hänggi K Bazargan S Onimus A Kasprzak A. TIM-3 blockade enhances IL-12-dependent antitumor immunity by promoting CD8(+) T cell and XCR1(+) dendritic cell spatial co-localization. J Immunother Cancer. (2022) 10:e003571. doi: 10.1136/jitc-2021-003571, PMID: 34987021 PMC8734033

[81] Sharonov GV Serebrovskaya EO Yuzhakova DV Britanova OV Chudakov DM. B cells, plasma cells and antibody repertoires in the tumour microenvironment. Nat Rev Immunol. (2020) 20:294–307. doi: 10.1038/s41577-019-0257-x, PMID: 31988391

[82] Biswas S Mandal G Payne KK Anadon CM Gatenbee CD Chaurio RA. IgA transcytosis and antigen recognition govern ovarian cancer immunity. Nature. (2021) 591:464–70. doi: 10.1038/s41586-020-03144-0, PMID: 33536615 PMC7969354

[83] Chen J Tan Y Sun F Hou L Zhang C Ge T. Single-cell transcriptome and antigen-immunoglobin analysis reveals the diversity of B cells in non-small cell lung cancer. Genome Biol. (2020) 21:152. doi: 10.1186/s13059-020-02064-6, PMID: 32580738 PMC7315523

[84] Wennhold K Thelen M Lehmann J Schran S Preugszat E Garcia-Marquez M. CD86+ Antigen-presenting B cells are increased in cancer, localize in tertiary lymphoid structures, and induce specific T-cell responses. Cancer Immunol Res. (2021) 9:1098–108. doi: 10.1158/2326-6066.CIR-20-0949, PMID: 34155067

[85] Sagiv-Barfi I Czerwinski DK Shree T Lohmeyer JJK Levy R. Intratumoral immunotherapy relies on B and T cell collaboration. Sci Immunol. (2022) 7:eabn5859. doi: 10.1126/sciimmunol.abn5859, PMID: 35622903 PMC9254330

[86] Wang Y Jia J Wang F Fang Y Yang Y Zhou Q. Pre-metastatic niche: formation, characteristics and therapeutic implication. Signal Transduct Target Ther. (2024) 9:236. doi: 10.1038/s41392-024-01937-7, PMID: 39317708 PMC11422510

[87] Ji H Hu C Yang X Liu Y Ji G Ge S. Lymph node metastasis in cancer progression: molecular mechanisms, clinical significance and therapeutic interventions. Signal Transduct Target Ther. (2023) 8:367. doi: 10.1038/s41392-023-01576-4, PMID: 37752146 PMC10522642

[88] Li L Shirkey MW Zhang T Piao W Li X Zhao J. Lymph node fibroblastic reticular cells preserve a tolerogenic niche in allograft transplantation through laminin α4. J Clin Invest. (2022) 132:e156994. doi: 10.1172/JCI156994, PMID: 35775481 PMC9246384

[89] du Bois H Heim TA Lund AW. Tumor-draining lymph nodes: At the crossroads of metastasis and immunity. Sci Immunol. (2021) 6:eabg3551. doi: 10.1126/sciimmunol.abg3551, PMID: 34516744 PMC8628268

[90] Rovera C Berestjuk I Lecacheur M Tavernier C Diazzi S Pisano S. Secretion of IL1 by dedifferentiated melanoma cells inhibits JAK1-STAT3-driven actomyosin contractility of lymph node fibroblastic reticular cells. Cancer Res. (2022) 82:1774–88. doi: 10.1158/0008-5472.CAN-21-0501, PMID: 35502542

[91] Chen S Zhu G Yang Y Wang F Xiao YT Zhang N. Single-cell analysis reveals transcriptomic remodellings in distinct cell types that contribute to human prostate cancer progression. Nat Cell Biol. (2021) 23:87–98. doi: 10.1038/s41556-020-00613-6, PMID: 33420488

[92] Deng J Liu Y Lee H Herrmann A Zhang W Zhang C. S1PR1-STAT3 signaling is crucial for myeloid cell colonization at future metastatic sites. Cancer Cell. (2012) 21:642–54. doi: 10.1016/j.ccr.2012.03.039, PMID: 22624714 PMC3360884

[93] van Pul KM Vuylsteke RJCLM van de Ven R Te Velde EA Rutgers EJT van den Tol PM. Selectively hampered activation of lymph node-resident dendritic cells precedes profound T cell suppression and metastatic spread in the breast cancer sentinel lymph node. J Immunother Cancer. (2019) 7:133. doi: 10.1186/s40425-019-0605-1, PMID: 31118093 PMC6530094

[94] Matsuura K Yamaguchi Y Ueno H Osaki A Arihiro K Toge T. Maturation of dendritic cells and T-cell responses in sentinel lymph nodes from patients with breast carcinoma. Cancer. (2006) 106:1227–36. doi: 10.1002/cncr.21729, PMID: 16475148

[95] Ogawa F Amano H Eshima K Ito Y Matsui Y Hosono K. Prostanoid induces premetastatic niche in regional lymph nodes. J Clin Invest. (2014) 124:4882–94. doi: 10.1172/JCI73530, PMID: 25271626 PMC4347225

[96] Go Y Tanaka H Tokumoto M Sakurai K Toyokawa T Kubo N. Tumor-associated macrophages extend along lymphatic flow in the pre-metastatic lymph nodes of human gastric cancer. Ann Surg Oncol. (2016) 23 Suppl 2:S230–5. doi: 10.1245/s10434-015-4458-7, PMID: 25743331

[97] Morrissey SM Zhang F Ding C Montoya-Durango DE Hu X Yang C. Tumor-derived exosomes drive immunosuppressive macrophages in a pre-metastatic niche through glycolytic dominant metabolic reprogramming. Cell Metab. (2021) 33:2040–2058.e10. doi: 10.1016/j.cmet.2021.09.002, PMID: 34559989 PMC8506837

[98] Coffelt SB Kersten K Doornebal CW Weiden J Vrijland K Hau CS. IL-17-producing γδ T cells and neutrophils conspire to promote breast cancer metastasis. Nature. (2015) 522:345–8. doi: 10.1038/nature14282, PMID: 25822788 PMC4475637

[99] Su X Brassard A Bartolomucci A Dhoparee-Doomah I Qiu Q Tsering T. Tumour extracellular vesicles induce neutrophil extracellular traps to promote lymph node metastasis. J Extracell Vesicles. (2023) 12:e12341. doi: 10.1002/jev2.12341, PMID: 37563798 PMC10415595

[100] Yaddanapudi K Stamp BF Subrahmanyam PB Smolenkov A Waigel SJ Gosain R. Single-cell immune mapping of melanoma sentinel lymph nodes reveals an actionable immunotolerant microenvironment. Clin Cancer Res. (2022) 28:2069–81. doi: 10.1158/1078-0432.CCR-21-0664, PMID: 35046061 PMC9840851

[101] Liu ZL Meng XY Bao RJ Shen MY Sun JJ Chen WD. Single cell deciphering of progression trajectories of the tumor ecosystem in head and neck cancer. Nat Commun. (2024) 15:2595. doi: 10.1038/s41467-024-46912-6, PMID: 38519500 PMC10959966

[102] Rahim MK Okholm TLH Jones KB McCarthy EE Liu CC Yee JL. Dynamic CD8(+) T cell responses to cancer immunotherapy in human regional lymph nodes are disrupted in metastatic lymph nodes. Cell. (2023) 186:1127–43.e18. doi: 10.1016/j.cell.2023.02.021, PMID: 36931243 PMC10348701

[103] Oliveira G Stromhaug K Cieri N Iorgulescu JB Klaeger S Wolff JO. Landscape of helper and regulatory antitumour CD4+ T cells in melanoma. Nature. (2022) 605:532–8. doi: 10.1038/s41586-022-04682-5, PMID: 35508657 PMC9815755

[104] Cachot A Bilous M Liu Y-C Li X Saillard M Cenerenti M. Tumor-specific cytolytic CD4 T cells mediate immunity against human cancer. Sci Adv. (2021) 7:eabe3348. doi: 10.1126/sciadv.abe3348, PMID: 33637530 PMC7909889

[105] Alonso R Flament H Lemoine S Sedlik C Bottasso E Péguillet I. Induction of anergic or regulatory tumor-specific CD4+ T cells in the tumor-draining lymph node. Nat Commun. (2018) 9:2113. doi: 10.1038/s41467-018-04524-x, PMID: 29844317 PMC5974295

[106] Kos K Aslam MA van de Ven R Wellenstein MD Pieters W van Weverwijk A. Tumor-educated Tregs drive organ-specific metastasis in breast cancer by impairing NK cells in the lymph node niche. Cell Rep. (2022) 38:110447. doi: 10.1016/j.celrep.2022.110447, PMID: 35235800

[107] Reticker-Flynn NE Zhang W Belk JA Basto PA Escalante NK Pilarowski GOW. Lymph node colonization induces tumor-immune tolerance to promote distant metastasis. Cell. (2022) 185:1924–42.e23. doi: 10.1016/j.cell.2022.04.019, PMID: 35525247 PMC9149144

[108] Núñez NG Tosello Boari J Ramos RN Richer W Cagnard N Anderfuhren CD. Tumor invasion in draining lymph nodes is associated with Treg accumulation in breast cancer patients. Nat Commun. (2020) 11:3272. doi: 10.1038/s41467-020-17046-2, PMID: 32601304 PMC7324591

[109] Lei P-J Pereira ER Andersson P Amoozgar Z Van Wijnbergen JW O’Melia MJ. Cancer cell plasticity and MHC-II-mediated immune tolerance promote breast cancer metastasis to lymph nodes. J Exp Med. (2023) 220:e20221847. doi: 10.1084/jem.20221847, PMID: 37341991 PMC10286805

[110] Hildner K Edelson BT Purtha WE Diamond M Matsushita H Kohyama M. Batf3 deficiency reveals a critical role for CD8alpha+ dendritic cells in cytotoxic T cell immunity. Science. (2008) 322:1097–100. doi: 10.1126/science.1164206, PMID: 19008445 PMC2756611

[111] Caronni N Simoncello F Stafetta F Guarnaccia C Ruiz-Moreno JS Opitz B. Downregulation of membrane trafficking proteins and lactate conditioning determine loss of dendritic cell function in lung cancer. Cancer Res. (2018) 78:1685–99. doi: 10.1158/0008-5472.CAN-17-1307, PMID: 29363545

[112] van den Hout MFCM Koster BD Sluijter BJR Molenkamp BG van de Ven R van den Eertwegh AJM. Melanoma sequentially suppresses different DC subsets in the sentinel lymph node, affecting disease spread and recurrence. Cancer Immunol Res. (2017) 5:969–77. doi: 10.1158/2326-6066.CIR-17-0110, PMID: 28935649

[113] Schenkel JM Herbst RH Canner D Li A Hillman M Shanahan S-L. Conventional type I dendritic cells maintain a reservoir of proliferative tumor-antigen specific TCF-1+ CD8+ T cells in tumor-draining lymph nodes. Immunity. (2021) 54:2338–2353.e6. doi: 10.1016/j.immuni.2021.08.026, PMID: 34534439 PMC8604155

[114] Bod L Kye Y-C Shi J Torlai Triglia E Schnell A Fessler J. B-cell-specific checkpoint molecules that regulate anti-tumour immunity. Nature. (2023) 619:348–56. doi: 10.1038/s41586-023-06231-0, PMID: 37344597 PMC10795478

[115] Piersiala K Hjalmarsson E da Silva PFN Lagebro V Kolev A Starkhammar M. Regulatory B cells producing IL-10 are increased in human tumor draining lymph nodes. Int J Cancer. (2023) 153:854–66. doi: 10.1002/ijc.34555, PMID: 37144812

[116] Mehdipour F Razmkhah M Hosseini A Bagheri M Safaei A Talei AR. Increased B regulatory phenotype in non-metastatic lymph nodes of node-positive breast cancer patients. Scand J Immunol. (2016) 83:195–202. doi: 10.1111/sji.12407, PMID: 26708831

[117] Ecker BL Kaur A Douglass SM Webster MR Almeida FV Marino GE. Age-related changes in HAPLN1 increase lymphatic permeability and affect routes of melanoma metastasis. Cancer Discov. (2019) 9:82–95. doi: 10.1158/2159-8290.CD-18-0168, PMID: 30279172 PMC6328344

[118] Leary N Walser S He Y Cousin N Pereira P Gallo A. Melanoma-derived extracellular vesicles mediate lymphatic remodelling and impair tumour immunity in draining lymph nodes. J Extracell Vesicles. (2022) 11:e12197. doi: 10.1002/jev2.12197, PMID: 35188342 PMC8859913

[119] Cousin N Cap S Dihr M Tacconi C Detmar M Dieterich LC. Lymphatic PD-L1 expression restricts tumor-specific CD8(+) T-cell responses. Cancer Res. (2021) 81:4133–44. doi: 10.1158/0008-5472.CAN-21-0633, PMID: 34099493 PMC9398148

[120] Li Y-L Chen C-H Chen J-Y Lai Y-S Wang S-C Jiang S-S. Single-cell analysis reveals immune modulation and metabolic switch in tumor-draining lymph nodes. Oncoimmunology. (2020) 9:1830513. doi: 10.1080/2162402X.2020.1830513, PMID: 33117603 PMC7575008

[121] Pelon F Bourachot B Kieffer Y Magagna I Mermet-Meillon F Bonnet I. Cancer-associated fibroblast heterogeneity in axillary lymph nodes drives metastases in breast cancer through complementary mechanisms. Nat Commun. (2020) 11:404. doi: 10.1038/s41467-019-14134-w, PMID: 31964880 PMC6972713

[122] Brighton PJ Szekeres PG Willars GB. Neuromedin U and its receptors: structure, function, and physiological roles. Pharmacol Rev. (2004) 56:231–48. doi: 10.1124/pr.56.2.3, PMID: 15169928

[123] Howard AD Wang R Pong SS Mellin TN Strack A Guan XM. Identification of receptors for neuromedin U and its role in feeding. Nature. (2000) 406:70–4. doi: 10.1038/35017610, PMID: 10894543

[124] Cardoso V Chesné J Ribeiro H García-Cassani B Carvalho T Bouchery T. Neuronal regulation of type 2 innate lymphoid cells via neuromedin U. Nature. (2017) 549:277–81. doi: 10.1038/nature23469, PMID: 28869974 PMC5714273

[125] Wallrapp A Riesenfeld SJ Burkett PR Abdulnour RE Nyman J Dionne D. The neuropeptide NMU amplifies ILC2-driven allergic lung inflammation. Nature. (2017) 549:351–6. doi: 10.1038/nature24029, PMID: 28902842 PMC5746044

[126] Moriyama M Sato T Inoue H Fukuyama S Teranishi H Kangawa K. The neuropeptide neuromedin U promotes inflammation by direct activation of mast cells. J Exp Med. (2005) 202:217–24. doi: 10.1084/jem.20050248, PMID: 16009716 PMC2213011

[127] Matsuo Y Yanase Y Irifuku R Takahagi S Mihara S Ishii K. Neuromedin U directly induces degranulation of skin mast cells, presumably via MRGPRX2. Allergy. (2018) 73:2256–60. doi: 10.1111/all.13555, PMID: 29987892

[128] Rosenfeld MG Mermod JJ Amara SG Swanson LW Sawchenko PE Rivier J. Production of a novel neuropeptide encoded by the calcitonin gene via tissue-specific RNA processing. Nature. (1983) 304:129–35. doi: 10.1038/304129a0, PMID: 6346105

[129] Pinho-Ribeiro FA Deng L Neel DV Erdogan O Basu H Yang D. Bacteria hijack a meningeal neuroimmune axis to facilitate brain invasion. Nature. (2023) 615:472–81. doi: 10.1038/s41586-023-05753-x, PMID: 36859544 PMC10593113

[130] Lu YZ Nayer B Singh SK Alshoubaki YK Yuan E Park AJ. CGRP sensory neurons promote tissue healing via neutrophils and macrophages. Nature. (2024) 628:604–11. doi: 10.1038/s41586-024-07237-y, PMID: 38538784 PMC11023938

[131] Baral P Umans BD Li L Wallrapp A Bist M Kirschbaum T. Nociceptor sensory neurons suppress neutrophil and γδ T cell responses in bacterial lung infections and lethal pneumonia. Nat Med. (2018) 24:417–26. doi: 10.1038/nm.4501, PMID: 29505031 PMC6263165

[132] Yang D Jacobson A Meerschaert KA Sifakis JJ Wu M Chen X. Nociceptor neurons direct goblet cells via a CGRP-RAMP1 axis to drive mucus production and gut barrier protection. Cell. (2022) 185:4190–205.e25. doi: 10.1016/j.cell.2022.09.024, PMID: 36243004 PMC9617795

[133] Okano M Hirahara K Kiuchi M Onoue M Iwamura C Kokubo K. Interleukin-33-activated neuropeptide CGRP-producing memory Th2 cells cooperate with somatosensory neurons to induce conjunctival itch. Immunity. (2022) 55:2352–68.e7. doi: 10.1016/j.immuni.2022.09.016, PMID: 36272417

[134] Russo AF Hay DL. CGRP physiology, pharmacology, and therapeutic targets: migraine and beyond. Physiol Rev. (2023) 103:1565–644. doi: 10.1152/physrev.00059.2021, PMID: 36454715 PMC9988538

[135] Walker CS Conner AC Poyner DR Hay DL. Regulation of signal transduction by calcitonin gene-related peptide receptors. Trends Pharmacol Sci. (2010) 31:476–83. doi: 10.1016/j.tips.2010.06.006, PMID: 20633935

[136] Nagashima H Mahlakõiv T Shih HY Davis FP Meylan F Huang Y. Neuropeptide CGRP limits group 2 innate lymphoid cell responses and constrains type 2 inflammation. Immunity. (2019) 51:682–95.e6. doi: 10.1016/j.immuni.2019.06.009, PMID: 31353223 PMC6801073

[137] Cohen JA Edwards TN Liu AW Hirai T Jones MR Wu J. Cutaneous TRPV1(+) neurons trigger protective innate type 17 anticipatory immunity. Cell. (2019) 178:919–32.e14. doi: 10.1016/j.cell.2019.06.022, PMID: 31353219 PMC6788801

[138] Crosson T Talbot S. Decoding nociceptor-DC dialogues. Immunity. (2023) 56:906–8. doi: 10.1016/j.immuni.2023.04.016, PMID: 37163991

[139] Fattori V Zaninelli TH Rasquel-Oliveira FS Heintz OK Jain A Sun L. Nociceptor-to-macrophage communication through CGRP/RAMP1 signaling drives endometriosis-associated pain and lesion growth in mice. Sci Transl Med. (2024) 16:eadk8230. doi: 10.1126/scitranslmed.adk8230, PMID: 39504351

[140] Murdock MH Yang CY Sun N Pao PC Blanco-Duque C Kahn MC. Multisensory gamma stimulation promotes glymphatic clearance of amyloid. Nature. (2024) 627:149–56. doi: 10.1038/s41586-024-07132-6, PMID: 38418876 PMC10917684

[141] Kubota Y Petras RE Ottaway CA Tubbs RR Farmer RG Fiocchi C. Colonic vasoactive intestinal peptide nerves in inflammatory bowel disease. Gastroenterology. (1992) 102:1242–51. doi: 10.1016/0016-5085(92)90762-N, PMID: 1551531

[142] Talbot J Hahn P Kroehling L Nguyen H Li D Littman DR. Feeding-dependent VIP neuron-ILC3 circuit regulates the intestinal barrier. Nature. (2020) 579:575–80. doi: 10.1038/s41586-020-2039-9, PMID: 32050257 PMC7135938

[143] Aguilar D Zhu F Millet A Millet N Germano P Pisegna J. Sensory neurons regulate stimulus-dependent humoral immunity in mouse models of bacterial infection and asthma. Nat Commun. (2024) 15:8914. doi: 10.1038/s41467-024-53269-3, PMID: 39414787 PMC11484968

[144] Delgado M Pozo D Ganea D. The significance of vasoactive intestinal peptide in immunomodulation. Pharmacol Rev. (2004) 56:249–90. doi: 10.1124/pr.56.2.7, PMID: 15169929

[145] Passang T Wang S Zhang H Zeng F Hsu PC Wang W. VPAC2 receptor signaling promotes growth and immunosuppression in pancreatic cancer. Cancer Res. (2024) 84:2954–67. doi: 10.1158/0008-5472.CAN-23-3628, PMID: 38809694 PMC11458156

[146] Ravindranathan S Passang T Li J-M Wang S Dhamsania R Ware MB. Targeting vasoactive intestinal peptide-mediated signaling enhances response to immune checkpoint therapy in pancreatic ductal adenocarcinoma. Nat Commun. (2022) 13:6418. doi: 10.1038/s41467-022-34242-4, PMID: 36302761 PMC9613684

[147] Frye BC Meiss F von Bubnoff D Zissel G Müller-Quernheim J. Vasoactive intestinal peptide in checkpoint inhibitor-induced pneumonitis. N Engl J Med. (2020) 382:2573–4. doi: 10.1056/NEJMc2000343, PMID: 32579820

[148] Vetrini F Brunetti-Pierri N Palmer DJ Bertin T Grove NC Finegold MJ. Vasoactive intestinal peptide increases hepatic transduction and reduces innate immune response following administration of helper-dependent Ad. Mol Ther. (2010) 18:1339–45. doi: 10.1038/mt.2010.84, PMID: 20461064 PMC2911263

[149] Perner C Flayer CH Zhu X Aderhold PA Dewan ZNA Voisin T. Substance P release by sensory neurons triggers dendritic cell migration and initiates the type-2 immune response to allergens. Immunity. (2020) 53:1063–77.e7. doi: 10.1016/j.immuni.2020.10.001, PMID: 33098765 PMC7677179

[150] Larsson O Tengroth L Xu Y Uddman R Kumlien Georén S Cardell LO. Substance P represents a novel first-line defense mechanism in the nose. J Allergy Clin Immunol. (2018) 141:128–36.e3. doi: 10.1016/j.jaci.2017.01.021, PMID: 28219705

[151] Ständer S Yosipovitch G. Substance P and neurokinin 1 receptor are new targets for the treatment of chronic pruritus. Br J Dermatol. (2019) 181:932–8. doi: 10.1111/bjd.18025, PMID: 31016733

[152] Serhan N Basso L Sibilano R Petitfils C Meixiong J Bonnart C. House dust mites activate nociceptor-mast cell clusters to drive type 2 skin inflammation. Nat Immunol. (2019) 20:1435–43. doi: 10.1038/s41590-019-0493-z, PMID: 31591569 PMC6858877

[153] Hollenhorst MI Nandigama R Evers SB Gamayun I Abdel Wadood N Salah A. Bitter taste signaling in tracheal epithelial brush cells elicits innate immune responses to bacterial infection. J Clin Invest. (2022) 132:e150951. doi: 10.1172/JCI150951, PMID: 35503420 PMC9246383

[154] Steinberg SF. Beta(1)-adrenergic receptor regulation revisited. Circ Res. (2018) 123:1199–201. doi: 10.1161/CIRCRESAHA.118.313884, PMID: 30571467 PMC6333418

[155] Guo Y Zhang X-N Su S Ruan Z-L Hu M-M Shu H-B. β-adrenoreceptor-triggered PKA activation negatively regulates the innate antiviral response. Cell Mol Immunol. (2023) 20:175–88. doi: 10.1038/s41423-022-00967-x, PMID: 36600052 PMC9886936

[156] Schiller M Azulay-Debby H Boshnak N Elyahu Y Korin B Ben-Shaanan TL. Optogenetic activation of local colonic sympathetic innervations attenuates colitis by limiting immune cell extravasation. Immunity. (2021) 54:1022–36.e8. doi: 10.1016/j.immuni.2021.04.007, PMID: 33932356 PMC8116309

[157] Ben-Shaanan TL Schiller M Azulay-Debby H Korin B Boshnak N Koren T. Modulation of anti-tumor immunity by the brain’s reward system. Nat Commun. (2018) 9:2723. doi: 10.1038/s41467-018-05283-5, PMID: 30006573 PMC6045610

[158] van der Heijden C Groh L Keating ST Kaffa C Noz MP Kersten S. Catecholamines induce trained immunity in monocytes *in vitro* and *in vivo* . Circ Res. (2020) 127:269–83. doi: 10.1161/CIRCRESAHA.119.315800, PMID: 32241223

[159] Vasamsetti SB Florentin J Coppin E Stiekema LCA Zheng KH Nisar MU. Sympathetic neuronal activation triggers myeloid progenitor proliferation and differentiation. Immunity. (2018) 49:93–106.e7. doi: 10.1016/j.immuni.2018.05.004, PMID: 29958804 PMC6051926

[160] Baruch EN Nagarajan P Gleber-Netto FO Rao X Xie T Akhter S. Inflammation induced by tumor-associated nerves promotes resistance to anti-PD-1 therapy in cancer patients and is targetable by interleukin-6 blockade. Res Sq. (2023). doi: 10.21203/rs.3.rs-3161761/v1, PMID: 37503252 PMC10371163

[161] Xue M Zhu Y Jiang Y Han L Shi M Su R. Schwann cells regulate tumor cells and cancer-associated fibroblasts in the pancreatic ductal adenocarcinoma microenvironment. Nat Commun. (2023) 14:4600. doi: 10.1038/s41467-023-40314-w, PMID: 37524695 PMC10390497

[162] Mo R-J Han Z-D Liang Y-K Ye J-H Wu S-L Lin SX. Expression of PD-L1 in tumor-associated nerves correlates with reduced CD8+ tumor-associated lymphocytes and poor prognosis in prostate cancer. Int J Cancer. (2019) 144:3099–110. doi: 10.1002/ijc.32061, PMID: 30537104

[163] Dubeykovskaya Z Si Y Chen X Worthley DL Renz BW Urbanska AM. Neural innervation stimulates splenic TFF2 to arrest myeloid cell expansion and cancer. Nat Commun. (2016) 7:10517. doi: 10.1038/ncomms10517, PMID: 26841680 PMC4742920

[164] Sloan EK Priceman SJ Cox BF Yu S Pimentel MA Tangkanangnukul V. The sympathetic nervous system induces a metastatic switch in primary breast cancer. Cancer Res. (2010) 70:7042–52. doi: 10.1158/0008-5472.CAN-10-0522, PMID: 20823155 PMC2940980

[165] Zhu J Naulaerts S Boudhan L Martin M Gatto L Van den Eynde BJ. Tumour immune rejection triggered by activation of α2-adrenergic receptors. Nature. (2023) 618:607–15. doi: 10.1038/s41586-023-06110-8, PMID: 37286594

[166] Globig A-M Zhao S Roginsky J Maltez VI Guiza J Avina-Ochoa N. The β1-adrenergic receptor links sympathetic nerves to T cell exhaustion. Nature. (2023) 622:383–92. doi: 10.1038/s41586-023-06568-6, PMID: 37731001 PMC10871066

[167] Kobayashi H Iida T Ochiai Y Malagola E Zhi X White RA. Neuro-mesenchymal interaction mediated by a β2-adrenergic nerve growth factor feedforward loop promotes colorectal cancer progression. Cancer Discov. (2025) 15:202–26. doi: 10.1158/2159-8290.CD-24-0287, PMID: 39137067 PMC11729495

[168] Li G Jiang Y Tong H Liu J Jiang Z Zhao Y. Sciatic nerve stimulation enhances NK cell cytotoxicity through dopamine signaling and synergizes immunotherapy in triple-negative breast cancer. Drug Resist Updat. (2025) 79:101212. doi: 10.1016/j.drup.2025.101212, PMID: 39951881

[169] Balood M Ahmadi M Eichwald T Ahmadi A Majdoubi A Roversi K. Nociceptor neurons affect cancer immunosurveillance. Nature. (2022) 611:405–12. doi: 10.1038/s41586-022-05374-w, PMID: 36323780 PMC9646485

[170] Restaino AC Walz A Vermeer SJ Barr J Kovács A Fettig RR. Functional neuronal circuits promote disease progression in cancer. Sci Adv. (2023) 9:eade4443. doi: 10.1126/sciadv.ade4443, PMID: 37163587 PMC10171812

[171] Hou Y Lin B Xu T Jiang J Luo S Chen W. The neurotransmitter calcitonin gene-related peptide shapes an immunosuppressive microenvironment in medullary thyroid cancer. Nat Commun. (2024) 15:5555. doi: 10.1038/s41467-024-49824-7, PMID: 39030177 PMC11271530

[172] Mauffrey P Tchitchek N Barroca V Bemelmans A-P Firlej V Allory Y. Progenitors from the central nervous system drive neurogenesis in cancer. Nature. (2019) 569:672–8. doi: 10.1038/s41586-019-1219-y, PMID: 31092925

[173] Finlay JB Ireland AS Hawgood SB Reyes T Ko T Olsen RR. Olfactory neuroblastoma mimics molecular heterogeneity and lineage trajectories of small-cell lung cancer. Cancer Cell. (2024) 42:1086–1105.e13. doi: 10.1016/j.ccell.2024.05.003, PMID: 38788720 PMC11186085

[174] Lu R Fan C Shangguan W Liu Y Li Y Shang Y. Neurons generated from carcinoma stem cells support cancer progression. Signal Transduct Target Ther. (2017) 2:16036. doi: 10.1038/sigtrans.2016.36, PMID: 29263908 PMC5657421

[175] Amit M Takahashi H Dragomir MP Lindemann A Gleber-Netto FO Pickering CR. Loss of p53 drives neuron reprogramming in head and neck cancer. Nature. (2020) 578:449–54. doi: 10.1038/s41586-020-1996-3, PMID: 32051587 PMC9723538

[176] Ayala GE Dai H Powell M Li R Ding Y Wheeler TM. Cancer-related axonogenesis and neurogenesis in prostate cancer. Clin Cancer Res. (2008) 14:7593–603. doi: 10.1158/1078-0432.CCR-08-1164, PMID: 19047084

[177] Hayakawa Y Sakitani K Konishi M Asfaha S Niikura R Tomita H. Nerve growth factor promotes gastric tumorigenesis through aberrant cholinergic signaling. Cancer Cell. (2017) 31:21–34. doi: 10.1016/j.ccell.2016.11.005, PMID: 27989802 PMC5225031

[178] Renz BW Takahashi R Tanaka T Macchini M Hayakawa Y Dantes Z. β2 adrenergic-neurotrophin feedforward loop promotes pancreatic cancer. Cancer Cell. (2018) 33:75–90.e7. doi: 10.1016/j.ccell.2017.11.007, PMID: 29249692 PMC5760435

[179] Allen JK Armaiz-Pena GN Nagaraja AS Sadaoui NC Ortiz T Dood R. Sustained adrenergic signaling promotes intratumoral innervation through BDNF induction. Cancer Res. (2018) 78:3233–42. doi: 10.1158/0008-5472.CAN-16-1701, PMID: 29661830 PMC6004256

[180] Zhi X Wu F Qian J Ochiai Y Lian G Malagola E. Nociceptive neurons promote gastric tumour progression via a CGRP-RAMP1 axis. Nature. (2025) 640:802–10. doi: 10.1038/s41586-025-08591-1, PMID: 39972142 PMC13022952

[181] Xu Q Cao Y Kong F Liu J Chen X Zhao Y. Multiple cancer cell types release LIF and Gal3 to hijack neural signals. Cell Res. (2024) 34:345–54. doi: 10.1038/s41422-024-00946-z, PMID: 38467743 PMC11061112

[182] Pan Y Hysinger JD Barron T Schindler NF Cobb O Guo X. NF1 mutation drives neuronal activity-dependent initiation of optic glioma. Nature. (2021) 594:277–82. doi: 10.1038/s41586-021-03580-6, PMID: 34040258 PMC8346229

[183] Chen P Wang W Liu R Lyu J Zhang L Li B. Olfactory sensory experience regulates gliomagenesis via neuronal IGF1. Nature. (2022) 606:550–6. doi: 10.1038/s41586-022-04719-9, PMID: 35545672

[184] Venkatesh HS Johung TB Caretti V Noll A Tang Y Nagaraja S. Neuronal activity promotes glioma growth through neuroligin-3 secretion. Cell. (2015) 161:803–16. doi: 10.1016/j.cell.2015.04.012, PMID: 25913192 PMC4447122

[185] Fnu T Shi P Zhang W Chung SSW Damoci CB Fang Y. Sympathetic neurons promote small cell lung cancer through the β2-adrenergic receptor. Cancer Discov. (2025) 15:616–32. doi: 10.1158/2159-8290.CD-24-0718, PMID: 39513738 PMC11875942

[186] Qin J Liu J Wei Z Li X Chen Z Li J. Targeted intervention in nerve-cancer crosstalk enhances pancreatic cancer chemotherapy. Nat Nanotechnol. (2025) 20:311–24. doi: 10.1038/s41565-024-01803-1, PMID: 39496914

[187] Thiel V Renders S Panten J Dross N Bauer K Azorin D. Characterization of single neurons reprogrammed by pancreatic cancer. Nature. (2025) 640:1042–51. doi: 10.1038/s41586-025-08735-3, PMID: 39961335 PMC12018453

[188] Zhu P Lu T Chen Z Liu B Fan D Li C. 5-hydroxytryptamine produced by enteric serotonergic neurons initiates colorectal cancer stem cell self-renewal and tumorigenesis. Neuron. (2022) 110:2268–82.e4. doi: 10.1016/j.neuron.2022.04.024, PMID: 35550066

[189] Anastasaki C Mo J Chen JK Chatterjee J Pan Y Scheaffer SM. Neuronal hyperexcitability drives central and peripheral nervous system tumor progression in models of neurofibromatosis-1. Nat Commun. (2022) 13:2785. doi: 10.1038/s41467-022-30466-6, PMID: 35589737 PMC9120229

[190] Pongratz G Straub RH. Role of peripheral nerve fibres in acute and chronic inflammation in arthritis. Nat Rev Rheumatol. (2013) 9:117–26. doi: 10.1038/nrrheum.2012.181, PMID: 23147892

[191] Kureshi CT Dougan SK. Cytokines in cancer. Cancer Cell. (2025) 43:15–35. doi: 10.1016/j.ccell.2024.11.011, PMID: 39672170 PMC11841838

[192] Cook AD Christensen AD Tewari D McMahon SB Hamilton JA. Immune cytokines and their receptors in inflammatory pain. Trends Immunol. (2018) 39:240–55. doi: 10.1016/j.it.2017.12.003, PMID: 29338939

[193] Schweizerhof M Stösser S Kurejova M Njoo C Gangadharan V Agarwal N. Hematopoietic colony-stimulating factors mediate tumor-nerve interactions and bone cancer pain. Nat Med. (2009) 15:802–7. doi: 10.1038/nm.1976, PMID: 19525966

[194] Lee JH Torisu-Itakara H Cochran AJ Kadison A Huynh Y Morton DL. Quantitative analysis of melanoma-induced cytokine-mediated immunosuppression in melanoma sentinel nodes. Clin Cancer Res. (2005) 11:107–12. doi: 10.1158/1078-0432.107.11.1, PMID: 15671534

[195] Oetjen LK Mack MR Feng J Whelan TM Niu H Guo CJ. Sensory neurons co-opt classical immune signaling pathways to mediate chronic itch. Cell. (2017) 171:217–28.e13. doi: 10.1016/j.cell.2017.08.006, PMID: 28890086 PMC5658016

[196] Lamorte S Quevedo R Jin R Neufeld L Liu ZQ Ciudad MT. Lymph node macrophages drive immune tolerance and resistance to cancer therapy by induction of the immune-regulatory cytokine IL-33. Cancer Cell. (2025) 43:955–969.e10. doi: 10.1016/j.ccell.2025.02.017, PMID: 40054466 PMC12074877

[197] García-Silva S Benito-Martín A Nogués L Hernández-Barranco A Mazariegos MS Santos V. Melanoma-derived small extracellular vesicles induce lymphangiogenesis and metastasis through an NGFR-dependent mechanism. Nat Cancer. (2021) 2:1387–405. doi: 10.1038/s43018-021-00272-y, PMID: 34957415 PMC8697753

[198] Clancy JW D’Souza-Schorey C. Tumor-derived extracellular vesicles: multifunctional entities in the tumor microenvironment. Annu Rev Pathol. (2023) 18:205–29. doi: 10.1146/annurev-pathmechdis-031521-022116, PMID: 36202098 PMC10410237

[199] Madeo M Colbert PL Vermeer DW Lucido CT Cain JT Vichaya EG. Cancer exosomes induce tumor innervation. Nat Commun. (2018) 9:4284. doi: 10.1038/s41467-018-06640-0, PMID: 30327461 PMC6191452

[200] Zheng S Hu C Lin Q Li T Li G Tian Q. Extracellular vesicle-packaged PIAT from cancer-associated fibroblasts drives neural remodeling by mediating m5C modification in pancreatic cancer mouse models. Sci Transl Med. (2024) 16:eadi0178. doi: 10.1126/scitranslmed.adi0178, PMID: 39018369

[201] Jones D Wang Z Chen IX Zhang S Banerji R Lei P-J. Solid stress impairs lymphocyte infiltration into lymph-node metastases. Nat BioMed Eng. (2021) 5:1426–36. doi: 10.1038/s41551-021-00766-1, PMID: 34282290 PMC8678215

[202] Jafarnejad M Woodruff MC Zawieja DC Carroll MC Moore JE. Modeling lymph flow and fluid exchange with blood vessels in lymph nodes. Lymphat Res Biol. (2015) 13:234–47. doi: 10.1089/lrb.2015.0028, PMID: 26683026 PMC4685511

[203] Abdreshov SN Demchenko GA Yeshmukhanbet AN Yessenova MA Mankibaeva SA Atanbaeva GK. Morphofunctional alteration of mesenteric lymph nodes in the inflammation of the abdominal cavity. Biol (Basel). (2024) 13:166. doi: 10.3390/biology13030166, PMID: 38534436 PMC10967999

[204] Das S Sarrou E Podgrabinska S Cassella M Mungamuri SK Feirt N. Tumor cell entry into the lymph node is controlled by CCL1 chemokine expressed by lymph node lymphatic sinuses. J Exp Med. (2013) 210:1509–28. doi: 10.1084/jem.20111627, PMID: 23878309 PMC3727324

[205] Uguen A. Another case of pacinian corpuscle in a lymph node. Anat Rec (Hoboken). (2018) 301:561–2. doi: 10.1002/ar.23765, PMID: 29281859

[206] Harter P Sehouli J Lorusso D Reuss A Vergote I Marth C. A randomized trial of lymphadenectomy in patients with advanced ovarian neoplasms. N Engl J Med. (2019) 380:822–32. doi: 10.1056/NEJMoa1808424, PMID: 30811909

[207] Faries MB Thompson JF Cochran AJ Andtbacka RH Mozzillo N Zager JS. Completion dissection or observation for sentinel-node metastasis in melanoma. N Engl J Med. (2017) 376:2211–22. doi: 10.1056/NEJMoa1613210, PMID: 28591523 PMC5548388

[208] Gschwend JE Heck MM Lehmann J Rübben H Albers P Wolff JM. Extended versus limited lymph node dissection in bladder cancer patients undergoing radical cystectomy: survival results from a prospective, randomized trial. Eur Urol. (2019) 75:604–11. doi: 10.1016/j.eururo.2018.09.047, PMID: 30337060

[209] Giuliano AE Ballman KV McCall L Beitsch PD Brennan MB Kelemen PR. Effect of axillary dissection vs no axillary dissection on 10-year overall survival among women with invasive breast cancer and sentinel node metastasis: the ACOSOG Z0011 (Alliance) randomized clinical trial. JAMA. (2017) 318:918–26. doi: 10.1001/jama.2017.11470, PMID: 28898379 PMC5672806

[210] Saddawi-Konefka R O’Farrell A Faraji F Clubb L Allevato MM Jensen SM. Lymphatic-preserving treatment sequencing with immune checkpoint inhibition unleashes cDC1-dependent antitumor immunity in HNSCC. Nat Commun. (2022) 13:4298. doi: 10.1038/s41467-022-31941-w, PMID: 35879302 PMC9314425

[211] Song E Mao T Dong H Boisserand LSB Antila S Bosenberg M. VEGF-C-driven lymphatic drainage enables immunosurveillance of brain tumours. Nature. (2020) 577:689–94. doi: 10.1038/s41586-019-1912-x, PMID: 31942068 PMC7100608

[212] van den Hout MFCM Sluijter BJR Santegoets SJAM van Leeuwen PAM van den Tol MP van den Eertwegh AJM. Local delivery of CpG-B and GM-CSF induces concerted activation of effector and regulatory T cells in the human melanoma sentinel lymph node. Cancer Immunol Immunother. (2016) 65:405–15. doi: 10.1007/s00262-016-1811-z, PMID: 26935057 PMC4826413

